# What agro-input dealers know, sell and say to smallholder farmers about pesticides: a mystery shopping and KAP analysis in Uganda

**DOI:** 10.1186/s12940-021-00775-2

**Published:** 2021-09-01

**Authors:** Philipp Staudacher, Curdin Brugger, Mirko S. Winkler, Christian Stamm, Andrea Farnham, Ruth Mubeezi, Rik I. L. Eggen, Isabel Günther

**Affiliations:** 1 Eawag, Swiss Federal Institute of Aquatic Science and Technology, Überlandstrasse 133, 8600 Dübendorf, Switzerland; 2 Institute of Biogeochemistry and Pollutant Dynamics, CHN, Universitätsstrasse 16, ETH Zürich, 8092 Zürich, Switzerland; 3 Department of Epidemiology and Public Health, Swiss Tropical and Public Health Institute, Basel, Switzerland; 4 University of Basel, Basel, Switzerland; 5 School of Public Health, Makerere University, Kampala, Uganda; 6 Development Economics Group & Center for Development and Cooperation (NADEL), ETH Zürich, Clausiusstrasse 37, 8092 Zürich, Switzerland

**Keywords:** Attitude, Counterfeit, Highly-hazardous, Knowledge, Pesticide dealer, Practices, Registration, Retail, Risk communication, Smallholder

## Abstract

**Background:**

Pesticides can have negative effects on human and environmental health, especially when not handled as intended. In many countries, agro-input dealers sell pesticides to smallholder farmers and are supposed to provide recommendations on application and handling. This study investigates the role of agro-input dealers in transmitting safety information from chemical manufacturers to smallholder farmers, assesses the safety of their shops, what products they sell, and how agro-input dealers abide by laws and recommendations on best practices for preventing pesticide risk situations.

**Methods:**

Applying a mixed-methods approach, we studied agro-input dealers in Central and Western Uganda. Structured questionnaires were applied to understand agro-input dealers’ knowledge, attitude and practices on pesticides (*n* = 402). Shop layout (*n* = 392) and sales interaction (*n* = 236) were assessed through observations. Actual behavior of agro-input dealers when selling pesticides was revealed through mystery shopping with local farmers buying pesticides (*n* = 94).

**Results:**

While 97.0% of agro-input dealers considered advising customers their responsibility, only 26.6% of mystery shoppers received any advice from agro-input dealers when buying pesticides. 53.2% of products purchased were officially recommended. Sales interactions focused mainly on product choice and price. Agro-input dealers showed limited understanding of labels and active ingredients. Moreover, 25.0% of shops were selling repackaged products, while 10.5% sold unmarked or unlabeled products. 90.1% of shops were lacking safety equipment. Pesticides of World Health Organization toxicity class I and II were sold most frequently. Awareness of health effects seemed to be high, although agro-input dealers showed incomplete hygiene practices and were lacking infrastructure. One reason for these findings might be that only 55.7% of agro-input dealers held a certificate of competency on safe handling of pesticides and even fewer (5.7%) were able to provide a government-approved up-to-date license.

**Conclusion:**

The combination of interviews, mystery shopping and observations proved to be useful, allowing the comparison of stated and actual behavior. While agro-input dealers want to sell pesticides and provide the corresponding risk advice, their customers might receive neither the appropriate product nor sufficient advice on proper handling. In light of the expected increase in pesticide use, affordable, accessible and repeated pesticide training and shop inspections are indispensable.

**Supplementary Information:**

The online version contains supplementary material available at 10.1186/s12940-021-00775-2.

## Background

Pesticides can have negative effects on human and environmental health, especially when not handled as intended. Smallholder pesticide use is increasing in low- and middle-income countries, and is often practiced without personal protective equipment (PPE) [1–3]. Pesticide exposure can lead to acute symptoms like headache and respiratory distress, or chronic health effects, such as increased risk for cancer and cognitive health impairment [4, 5]. Examples of environmental effects include weakened honey bee immune systems, eggshell thinning in birds, and damage to reproductive systems among amphibians and mammals [6].

Agro-input dealers are small, often independent stockists or distributors of agricultural inputs, such as pesticides. The private retail sector, including agro-input dealers, is often the dominant source of pesticides for farmers in low- and middle-income countries [7]. Studies show that smallholders also consider agro-input dealers a major source of information for pest management [2, 8]. Pesticide manufacturers, on the other hand, do not have direct contact with agro-input dealers and farmers, and thus use written formats such as product labels to inform their customers [9]. The label on a pesticide container is intended to provide all relevant information on content and handling, as well as protective measures to be taken for the environment and human health [10]. Agro-input dealers are crucial in providing farmers access to products with sufficient labelling, translating and transmitting the necessary information (to often illiterate farmers) and providing access to recommended tools and protective equipment where necessary [11].

Despite agro-input dealers’ essential role in protecting humans and the environment from the harmful use of pesticides, only a few studies have investigated their knowledge, the safety of their shops, and the advice they give to their customers, including how they transmit safety recommendations from the chemical manufacturers to the users. Some studies from low- and middle-income countries suggest that agro-input dealers are not interested in providing proper advice, as this might reduce product sales [12, 13]. Other studies found that agro-input dealers are not properly trained [14, 15] and base their advice on knowledge gained through personal experience, brand ambassadors, and level of commission [16, 17]. On the other hand, many studies suggest that farmers take the advice from agro-input dealers seriously and adopt the suggested practices [18–20]. The role of agro-input dealers in pesticide risk advice is underlined by the fact that farmers often prefer them as a source of information over alternatives such as extension services [21] due to closer proximity and higher accessibility [14]. Unfortunately, the licensed shop owners are regularly absent from their agro-input shops and employ untrained staff instead, thus making proper customer advice difficult [15, 22].

Despite the abundance of agro-input shops selling potentially harmful chemicals, little is known about the safety of the shops, the knowledge of the agro-input dealers and the advice given to farmers. To fill this gap we conducted a study among agro-input dealers in Uganda. Previous studies have investigated farmers’ pesticide use and related risks as well as information behavior in Uganda, identifying agro-input dealers as the primary provider of pesticides and an information source for smallholders on risk factors for safe pesticide use [2, 3, 8, 23]. This study investigated what pesticides agro-input dealers sold, what safety advice they gave to farmers, what they knew about pesticides and believed about the risks, and how they are abiding by the laws, recommended guidelines, and best practices to prevent pesticide risk situations in their own shops.

## Methods

**Fig. 1:**
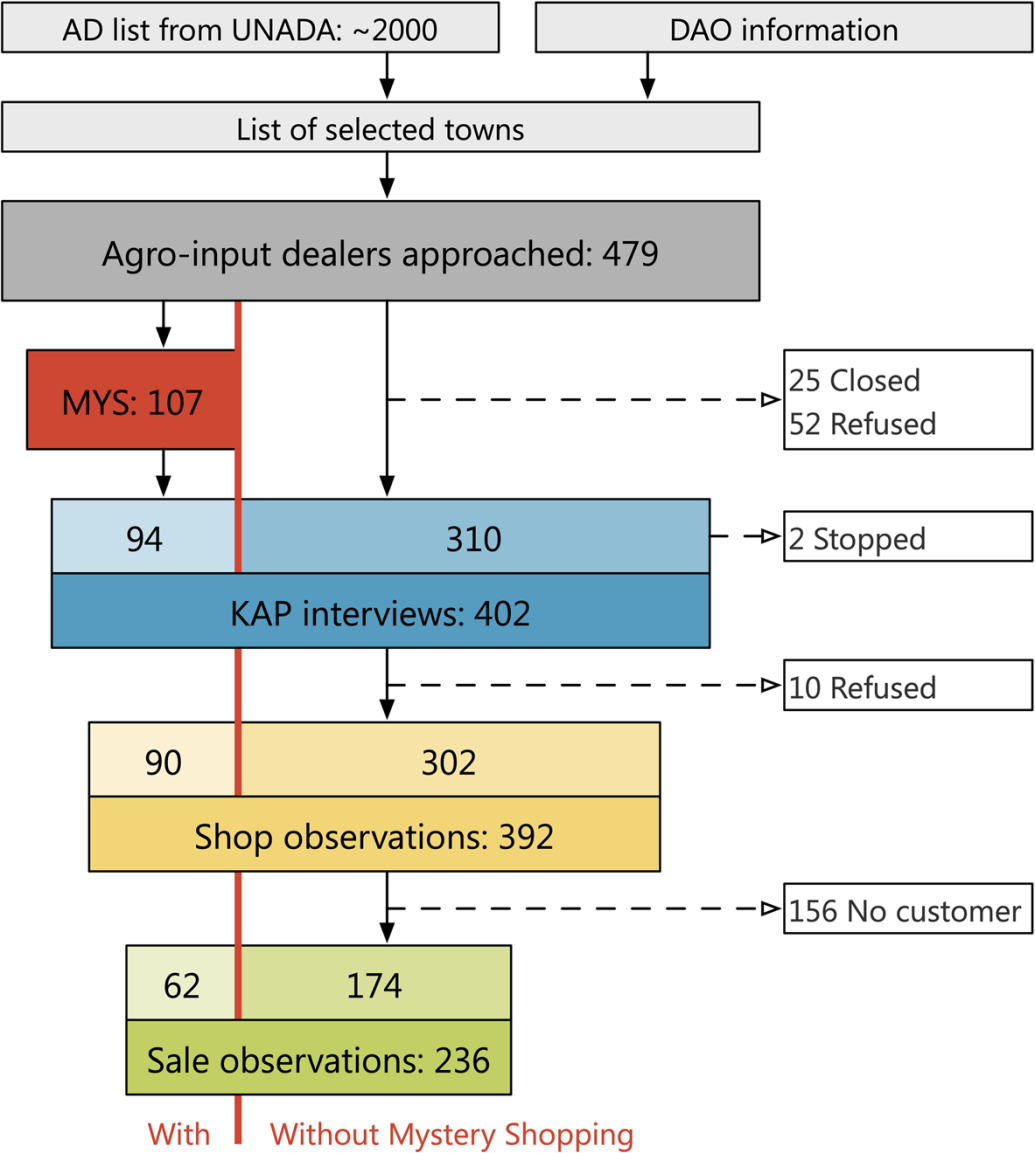
Distribution of study participants (own illustration, adapted from CONSORT flowchart [24]). AD: agro-input dealer; DAO: district agricultural officer. Reading explanation: Of the 479 agro-input dealers approached, 25 Shops were closed and 50 agro-input dealers refused to take part in the study, resulting in 404 KAP interviews (310 + 94) that were started. 402 of the interviews were finished and used for analysis. From 107 MYS conducted, ten MYS had to be excluded from analysis because the agro-input dealers refused to take part in the KAP survey. Additionally, three MYS could not be included in the analysis because the KAP survey was conducted with a different staff member than the MYS. 10 agro-input dealers refused the shop observation, thus 392 shop observations were conducted. At 236 shops a sale observation took place because 156 agro-input dealers did not have any customers during the time the researchers were at their store or they did not give consent. In one case, only a sale observation but no shop observation was conducted

To compare stated with actual behavior of agro-input dealers, this study combined three different data collection modules: i) mystery shopping (MYS) to observe agro-input dealers providing pesticide risk and safety advice to farmers through trained undercover observers; ii) knowledge, attitude and practice (KAP) interviews on safe pesticide use and handling with sales staff working in agro-input dealers shops, and iii) observations of shop premises and sales interactions**.** The KAP interview as well as sales and shop observations were conducted with the complete sample, while only a sub-sample of 25% was selected for a mystery shopping *before* the KAP interview (Fig. 1). KAP interviews were conducted with the same person who sold the pesticide during the mystery shopping.

### Study setting in Uganda

In order to be allowed to sell pesticides, agro-input dealers in Uganda are required to complete eleven years of school (ordinary secondary school, Senior Four certificate), complete a certification of competency on safe handling of pesticide (CCSP), and register their business with several Ugandan authorities [25]. The curriculum of the two-week long training course for the certification of competency on safe handling of pesticide contains the relevant information a pesticide dealer should know about. Pest identification and pest control measures (e.g. cultural control, integrated pest management) are as much part of the program as regulations, application practices, and equipment [26]. In 2009, a census in Uganda of 2064 agro-input dealers found that only a minority had not completed mandatory school (12%), while less than half (45%) reported undergoing training for a certification of competency on safe handling of pesticide. However, 31% reported an academic specialization in the field of agriculture. The majority reported a trading license (85%), while only 27% were registered with the Ministry of Agriculture, Animal Industry and Fisheries [27].

### Sample

**Fig. 2:**
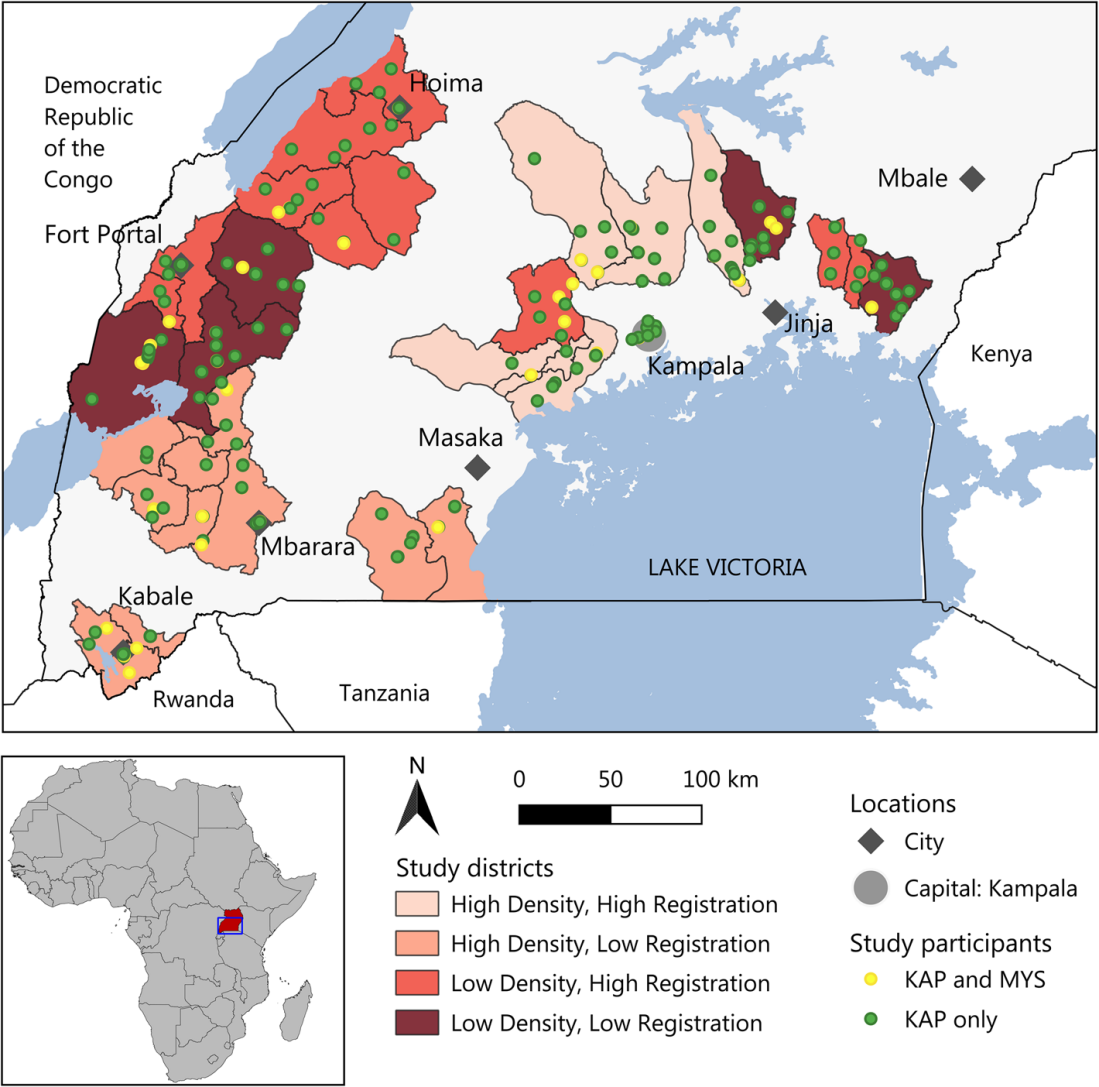
Map of Uganda showing the 35 selected districts and corresponding study sites. A green point indicates that an agro-input dealer shop was identified and a KAP interview conducted. A yellow point indicates, that a KAP took place with a MYS in the same shop beforehand

The study was conducted in 35 districts and 146 towns in the central and western region of Uganda in October and November 2019 (Fig. 2). To ensure a representative sample, districts with high and low agro-input dealer density (estimated number of agro-input dealers per agricultural household from the corresponding official national agricultural census [28]), and with high and low number of registered agro-input dealers (share of self-reported registered agro-input dealers according to the first and only national agro-input dealer census [27]) were selected. Because of logistical considerations only districts with a majority of the population speaking Luganda or Runyankore were included. To ensure at least one open agro-input shop per town was present, the focus was placed on larger towns. In each town agro-input dealers were selected by a predefined process using a coin toss to maximize random selection.

The agro-input dealer census from 2009 identified 1588 agro-input dealers in the central and western region of Uganda [27]. Across the 35 districts and 146 towns we approached 479 agro-input dealers to reach the target sample size of 400 agro-input dealers, representing approximately 25% of the 2009 agro-input dealer population. The KAP interviews and shop observations were conducted in 146 towns (median: 7 per district), while the additional mystery shopping was conducted in 65 towns (median: 3 per district) (Fig. 1). To ensure that the final sample was representative of the cultural and climatic context of central and western Uganda, we practiced stratified sampling for important agro-input dealer characteristics, such as registration status and rural vs. urban settings.

### Data collection

Mystery shopping is a form of covert participatory observation to gain a better understanding of the interaction between a seller and a customer [29]. A mystery shopper who is trained by the researcher enters a store and acts as a typical customer in need of a product or service. After acquiring the product or service, the mystery shopper is interviewed by a researcher, through which important information on the services in the respective store is gained [16, 30]. Outside of market research and customer service evaluation, mystery shopping is not yet widely used. A few studies have successfully applied mystery shopping in public health settings in Europe [31, 32] and Africa: The studies in Kenya [33] and Tanzania [30, 34] investigated the drugs sold and advice provided by drug retailers when presented with symptoms by a mystery shopper.

For the mystery shopping technique to produce valid and reliable data it is important that the mystery shopper appears to be a plausible regular customer and that all mystery shoppers follow the same protocol. In this study, we recruited local farmers and systematically trained them to describe the case problem with the same four sentences. The case problem used was the fall armyworm affecting the farmer’s maize. The fall armyworm was first noted in Africa in 2014 and has become a devastating pest in sub-Saharan Africa, including Uganda [35, 36]. In the case where farmers were not given any advice before they had paid for the pesticide, they were instructed to ask three specific questions on health risks and protection. After completion of the mystery shopping the farmers were debriefed about their shopping experience and interaction with the agro-input dealers, using a standardized structured questionnaire in ODK (Open Data Kit) [37].

KAP interviews are a well-established method to collect a large amount of quantitative data from study participants on self-reported knowledge, attitude and practices related to a specific field [38]. In this study, KAP interviews were conducted with a standardized structured questionnaire in Luganda, Runyankore or English. The KAP survey covered knowledge, attitude and practices on their profession as agro-input dealers, handling and protection of pesticides, effects of pesticides on human and environmental health, alternatives to pesticide use, and general agricultural aspects. In addition, the interviews included questions on socio-demographics, education, training, sales experience, shop organization, and personal health.

In parallel with the KAP survey, each interviewer also conducted two observations per interviewee: i) the dealer’s sale interaction with a customer; ii) the shop premises regarding compliance with official safety recommendations [25, 39]. Both the sale interaction and the shop premises were studied through a non-participatory, structured and overt observation. Refusal to take part in one or both observations did not exclude the dealer from the study.

The research team was thoroughly trained for 10 days and conducted a pilot study in one district, Wakiso. The questionnaires were translated from English to Luganda and Runyankore by professional translators and refined after the pilot. Ethical clearance was obtained in Uganda and Switzerland (see declarations).

### Data and analysis

Descriptive statistics were estimated for all variables using R version 4.0.2 [40]. To assess whether the subsamples mystery shopping and sale observation were drawn from the same distribution as KAP, we conducted a two-sample Kolmogorov–Smirnov test for each numerical variable, while for categorical variables we applied the chi-square test to test whether subsets differed. All prices were calculated from Ugandan Shilling to United States Dollar ($), using the conversion rate of October 2019 at 1:3700.

The agro-input dealer shop observations were based on guidelines by both the Ministry of Agriculture, Animal Industry and Fisheries and the Food and Agriculture Organization of the United Nations [25, 39]. The failure to adhere to these recommendations was categorized into three increasing categories of seriousness, following the work of Akhabuhaya [41]: somewhat serious, serious and very serious. These categories were selected to reflect the risk for acute intoxication through oral or dermal exposure (very serious), chronic intoxication through inhalation, or dermal exposure (serious), or otherwise not following the guidelines (somewhat serious).

When comparing different pesticide products, their active ingredients, and their toxicity we use the toxicity classes recommended by the World Health Organization (WHO) [42]. The WHO classifies pesticides based on acute oral and dermal toxicity of the AI, defining five classes based on different LD_50_: Ia – extremely hazardous, Ib – highly hazardous, II – moderately hazardous, III – slightly hazardous, and U – unlikely to present acute hazard (formerly class IV – Less hazardous) (Supplementary Table ST 1, Additional File 1).

**Fig. 3:**
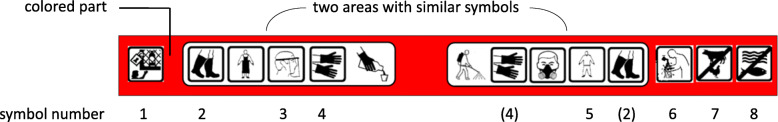
Example label used for knowledge test. The image was provided without the text and numbers, see also Supplementary Table ST 2, Additional File 1. Image adapted from FAO and WHO [9]

During the agro-input dealers’ knowledge assessment, the first set of questions referred to a typical safety label, which is normally placed on the bottom end of a pesticide container. The colored part, the two areas with similar symbols, as well as the individual symbols have different meanings and are supposed to be read (and understood) from left to right (Fig. 3).

For the attitude assessment, we adapted a battery of statements originally designed for smallholder vegetable farmers in Southeast Asia [43]. The statements were determined to be disputed, when the absolute difference between 50 and the share of answers stating ‘true’ or ‘yes’ was smaller than 20 (Example: 10% Yes, not disputed: |50–10| > 20; 35% Yes, disputed: |50–35| < 20).

Using a simple regression (linear for score-dependent variables and logistic for binomial dependent variables) we analysed the relationship between the independent variables age (years), education (years), sex (female/male), positon (owner/employee), having a certification of competency on safe handling of pesticide (yes/no), having a shop license (yes/no), distance to Kampala (rounded to 100 km), distance to next city (rounded to 100 km) and the following dependent variables: knowledge of pesticide labels, product use and alternatives (score 1–10), pesticide beliefs (score 1–16), hygiene practices, wearing gloves and repackaging of pesticides (score 1–12), very serious violation of laws or recommendations (yes/no), selling pesticides of class WHO I and II during mystery shopping (yes/no) and providing any advice during mystery shopping (yes/no).

The dataset, as well as the instruction materials and questionnaires from the collection are accessible under 10.25678/0004CX.

## Results

### Pesticide dealers

Of the 479 agro-input dealers approached, we sampled 107 shops for a mystery shopping, 402 for knowledge, attitude and practice interviews, 392 for shop observations, and 236 for sale observations (Fig. 1). The 402 agro-input dealers interviewed were close to thirty years old (28.5 years), and the majority were women (60.7%). Roughly half of them were shop owners (53.0%), and half of them employees (47.0%), with a median employment in agro-input dealer shops of three years (equaling also the median experience in selling pesticides). Agro-input dealers in Uganda worked on average twelve hours per day, seven days a week, and earned $54.1 per month (Table 1). The majority of agro-input dealers (83.3%) had completed mandatory school (seven years primary and four years secondary) or more. Of all agro-input dealers, 29.3% had a higher education in agriculture, veterinary, pharmacy or medicine, whereas 19.7% were trained in business, administration, accounting, etc. (Supplementary Table ST 3, Additional File 1).

**Table 1:** Sample description for agro-input dealers, their education and training

Agro-input dealers	Unit	KAP^a^	OBS^a^	MYS^a^
Number of participants	n	402	236	94
Female (vs male)	%	60.7	61.0	62.8
Age (median^b^)	years	28.5 (6.7)	29 (7.4)	29 (7.4)
Employees (vs owners)	%	47.0	44.5	43.6
Employment in this shop (median^b^)	years	3 (3.0)	3 (3.0)	3 (3.0)
Working hours per day (median^b^)	n	12 (1.5)	12 (1.5)	12 (1.5)
Working days per week (median^b^)	n	7 (0)	7 (0)	6 (0.7)
Monthly income from shop (median^b^)	$	54.1 (40.1)	54.1 (40.1)	54.1 (40.1)
Responsibilities around pesticides in the shop (multiple choice)				
Responsible for everything (see below)	%	76.1	74.2	75.5
Conducting sales	%	23.9	25.90	24.5
Giving advice	%	20.9	23.3	19.1
Handling pesticides	%	18.7	20.8	17.0
Bookkeeping	%	18.4	21.2	17.0
Cleaning	%	17.7	19.9	14.9
(Re)packaging	%	6.97	7.6	8.5
General Education (median^b^)	years	13 (3.0)	13 (3.0)	13 (3.0)
Experience selling pesticides (median^b^)	years	3 (3.0)	4 (3.0)	4 (3.0)
Interviewee has a CCSP^c^	%	55.7	64.8***	61.7
Ever received any training …				
on pesticides in general	%	77.9	74.6	76.6
in alternatives to pesticides	%	43.8	46.2	45.7
in pesticide application	%	90.3	90.7	88.3

Note: No significant differences were found with one exception: CCSP is different between KAP and OBS: ***Significant difference at p < 0.001

^*a*^*The samples are abbreviated with KAP for the full sample of interviewees, MYS for those participating in mystery shopping and OBS for those participating in the sales observation*

^*b*^*Median with median absolute deviation in parentheses*

^*c*^*CCSP: Certification of competency on safe handling of pesticide*

The majority of agro-input dealers (76.1%) were responsible for everything in the shop, while the others were mainly responsible for conducting sales (23.9%) and giving advice (20.9%) (Table 1). Only 55.7% of the interviewed agro-input dealers held a certification of competency on safe handling of pesticide, a requirement to sell pesticides in Uganda. But more than 90.3% had received instructions on pesticide application and 77.9% had received another general training on pesticides (Table 1). The contents of the general pesticide training were primarily safe use and handling of chemicals (86.9%) (Supplementary Table ST 4, Additional File 1) and were provided by either the Uganda National Agro-Input Dealers’ Association (UNADA), the shop owner, a government agency, or schools and university; agricultural extension services and pesticide manufacturers played a less important role. On the other hand, less than half of agro-input dealers had ever received training on alternatives to synthetic pesticides, while 38.1% of those agro-input dealers who had, received it in school or university (Supplementary Table ST 5, Additional File 1). The sample does not show indications of imbalances between the main sample for KAP and the subsamples for sales observations and mystery shopping.

### Pesticide shops

The majority of agro-input shops (82.3%) reported at least one employee with a certification of competency on safe handling of pesticide (Table 2) and had been inspected at least once in the past (81.1%), mostly to check for counterfeits or other unauthorized products (36.8%), or license approval or renewal (30.6%). A shop license issued by the Ministry of Agriculture, Animal Industry and Fisheries is mandatory to sell pesticides in Uganda. However, only 5.7% of shops could provide an up-to-date license, while 41.5% stated they had no license (Supplementary Table ST 6, Additional File 1). Each shop has a median estimate of 20 customers per day, half of which buy pesticides for a median price of $4.1. The customers are primarily smallholder farmers (90%), male (70%) with a median farm size of one acre (Table 2).

**Table 2:** Sample description for shops and customers

Shop organization and customer relations	Unit	KAP^a^	OBS^a^	MYS^a^
At least one person with CCSP^c^ working in shop	%	82.3	88.6***	87.2
Number of employees per shop (median^b^)	n	2 (1.5)	2 (1.5)	2 (1.5)
Sole ownership (vs partnerships or cooperatives)	%	87.8	89.0	87.2
Owner regularly interacting with customers	%	88.8	92.4*	92.6
At least one shop employee visiting farmer fields	%	68.7	69.9	63.8
Estimated shop size (median^b^)	m^2^	9 (7.4)	9 (7.4)	9 (7.4)
Shop age (median^b^)	years	4 (2.97)	4 (2.97)	4 (2.97)
Open days per week (median^b^)	n	7 (0)	7 (0)	6.5 (0.7)
Customers per day (median) ^b^	n	20 (14.8)	20 (14.8)	20 (14.8)
Number of pesticide transactions per day (median^b^)	n	10 (7.4)	10 (7.4)	10 (7.4)
Spending on pesticides per transaction (median^b^)	$	4.1 (4.0)	4.1 (4.0)	4.1 (4.0)
Number of customers per season (median^b^)	n	1680 (1068)	1920 (1423)	1680 (1328)
Number of competitors in parish (median^b^)	n	5 (4.5)	6 (4.5)	7 (5.9)
Share of non-smallholder customers (median^b^)	%	10 (14.8)	15 (19.3)	10 (14.8)
Share of female customers (median^b^)	%	30 (14.8)	30 (14.8)	30 (14.8)
Customer farm size (median^b^)	acre	1 (0.7)	1 (0.7)	1 (0.7)
Customers with smartphone (median share^b^)	%	20 (22.2)	20 (22.2)	25 (22.2)

Note: No significant differences were found with two exceptions: CCSP is different between KAP and OBS: ***Significant difference at p < 0.001 and owner interaction for OBS *Significant difference at p < 0.05

^*a*^*The samples are abbreviated with KAP for the full sample of interviewees, MYS for those participating in mystery shopping and OBS for those participating in the sales observation*

^*b*^*Median with median absolute deviation in parentheses*

^*c*^*CCSP: Certification of competency on safe handling of pesticide*

The shop observation revealed that 100% of shops showed *somewhat serious*, 98% of shops *serious*, and 36% *very serious* deviations from the shop setup recommended by both the Ministry of Agriculture, Animal Industry and Fisheries and Food and the Agriculture Organization of the United Nations [25, 39]. *Very serious* deviations were found in a quarter of shops that were repackaging pesticide containers (25.0%) and a tenth of shops that were using unmarked/unlabeled pesticide containers (10.5%). The *serious* deviations were lack of safety equipment (90.1%), such as PPE, water, soap, or materials for spill-cleanup such as brooms. Also prominent were obstructed fire exits (41.6%), insufficient ventilation (31.1%), and small shop sizes (41.1%). Moreover, *somewhat serious* deviations like the absence of safety displays (99.7%), missing firefighting equipment (93.4%), absence of documents (85.7%), or inadequate floor drainage (78.8%) were frequently observed (Supplementary Table ST 7, Additional File 1).

Agro-input dealers were commonly not using PPE when handling pesticides in the shop. The most accessible PPE (to more than 69%) were also the most used (by more than 30% of those who had access): Masks without carbon filter, long sleeved shirts, gloves, long pants, and rubber boots (Supplementary Fig. SF 1, Additional File 1). The reasons agro-input dealers gave as to why they weren’t using PPE were lack of availability (43.3%), high price (33.1%), lack of comfort (32.1%), and the belief that they weren’t needed (25.9%).

Proper hygiene practices are also relevant to minimizing risks. Nevertheless, 55.5% of agro-input dealers reported drinking beverages and 43.0% eating food in the shop. The majority (92.0%) claimed to wash their hands immediately after pesticide handling and 95.0% change their clothes after a day involving pesticide handling (Supplementary Table ST 8, Additional File 1).

Handling of pesticides and residues can be a source of risk: A third of agro-input dealers had ever opened sealed containers to sell smaller quantities in different containers (repackaging) and a quarter were currently doing it. Those who stopped did so because of health effects (53.8%) and illegality (28.2%). The most commonly repackaged active ingredients were mancozeb (54.0%) and glyphosate (25.0%). Agro-input dealers commonly did not dispose of returned pesticide containers at all (45.0%). Those who did, mostly burnt them (35.3%) or brought them to municipal disposal sites or other trash (11.7%) (Supplementary Table ST 9, Additional File 1).

### Pesticides on sale

Pesticides are the most sold product of agro-input dealers (88.6%) and the most profitable (80.5%) (Supplementary Table ST 10, Additional File 1). Specifically, the most sold products are herbicides (47.3%), insecticides (33.3%), and fungicides (8.0%), while the most profitable products are herbicides (50.7%), insecticides (22.9%), and fungicides (6.7%). Besides pesticides, most shops also sell fertilizers (92.3%), seeds (85.6%), and spray pumps (65.4%). The most commonly sold PPE in shops are gumboots (35.6%), followed by masks without carbon filter (31.6%), gloves (27.6%), masks with carbon filter (17.4%), and glasses (11.7%) (Supplementary Table ST 11, Additional File 1).

A look at the WHO toxicity class of the 15 bestselling pesticide brands according to the KAP interviews reveals that 26.5% of active ingredients were of class Ib and 47.6% of class II, so moderately to highly toxic. The only active ingredient of class III and U were the herbicide glyphosate and the fungicide mancozeb respectively. Shop observations revealed that the most common WHO toxicity class in shops was III (41.1%) (Supplementary Fig. SF 2, Additional File 1). Labels on pesticides in the shop are mostly available in English (91.2%). Only 19.8% of labels are available in a local language.

**Fig. 4:**
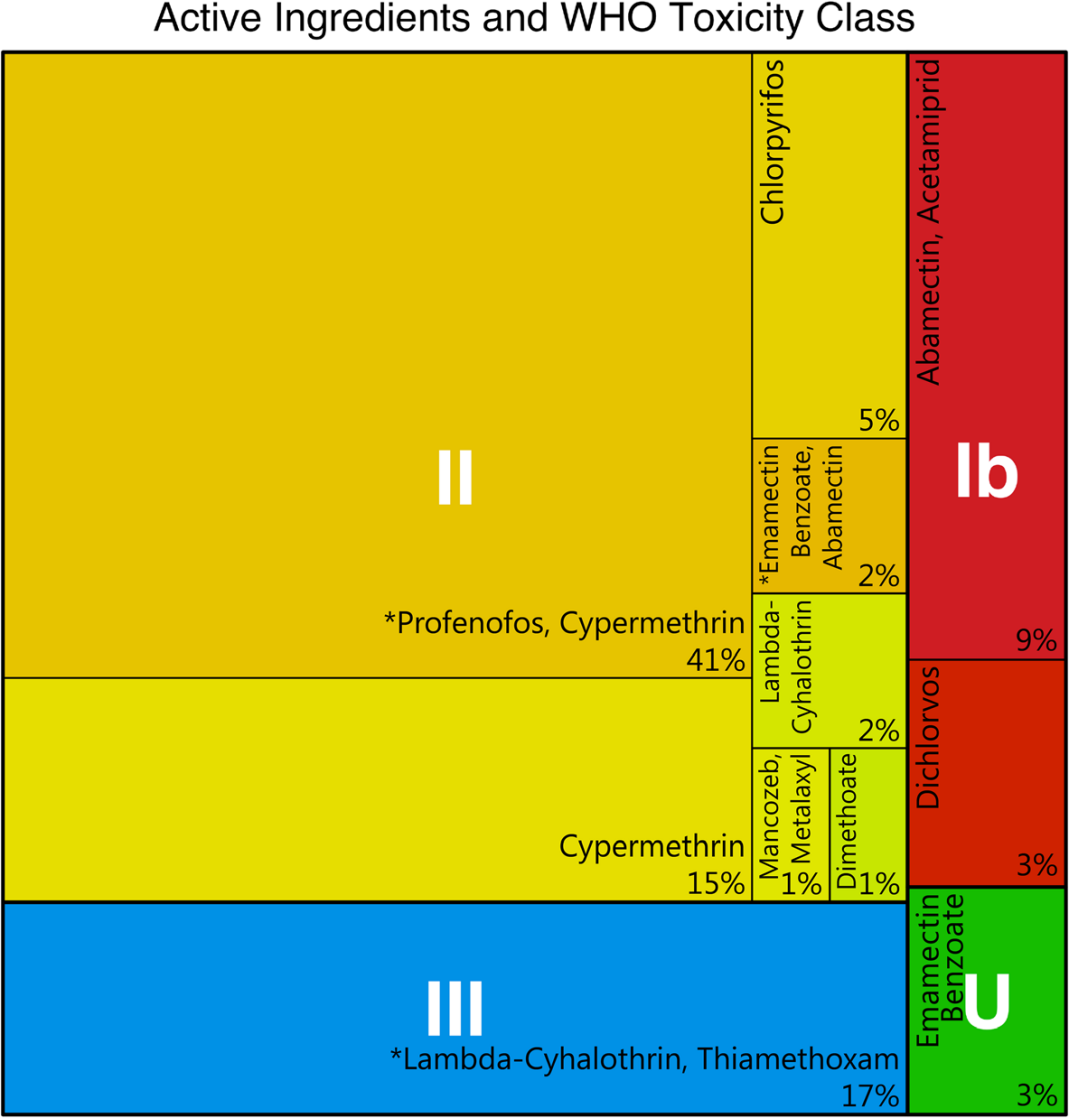
Active ingredients of purchased pesticides during mystery shopping and their WHO toxicity class. * = Active ingredient is on the list of approved pesticides against the fall army worm from the Ministry of Agriculture, Animal Industry and Fisheries

The 94 mystery shoppers purchased 25 different pesticide brands against the fall armyworm, consisting of eleven different active ingredient combinations. While only four of the brands were approved by the Ministry of Agriculture, Animal Industry and Fisheries for use against fall armyworm (Supplementary Fig. SF 3, Additional File 1), they made up 53% of purchases (Supplementary Table ST 12, Additional File 1). Of the products purchased, 13% were WHO toxicity class Ib and 68% were class II (Fig. 4). The most expensive pesticide products purchased were those in class U (*n* = 3, $1.85), followed by class Ib (*n* = 11, $1.69), class III ($1.56, *n* = 16), and class II (*n* = 64, mean = $1.44). Class II products were significantly less expensive than both class Ib (*p* = 0.018) and class U products (*p* = 0.047).

### Dealer advice

**Fig. 5:**
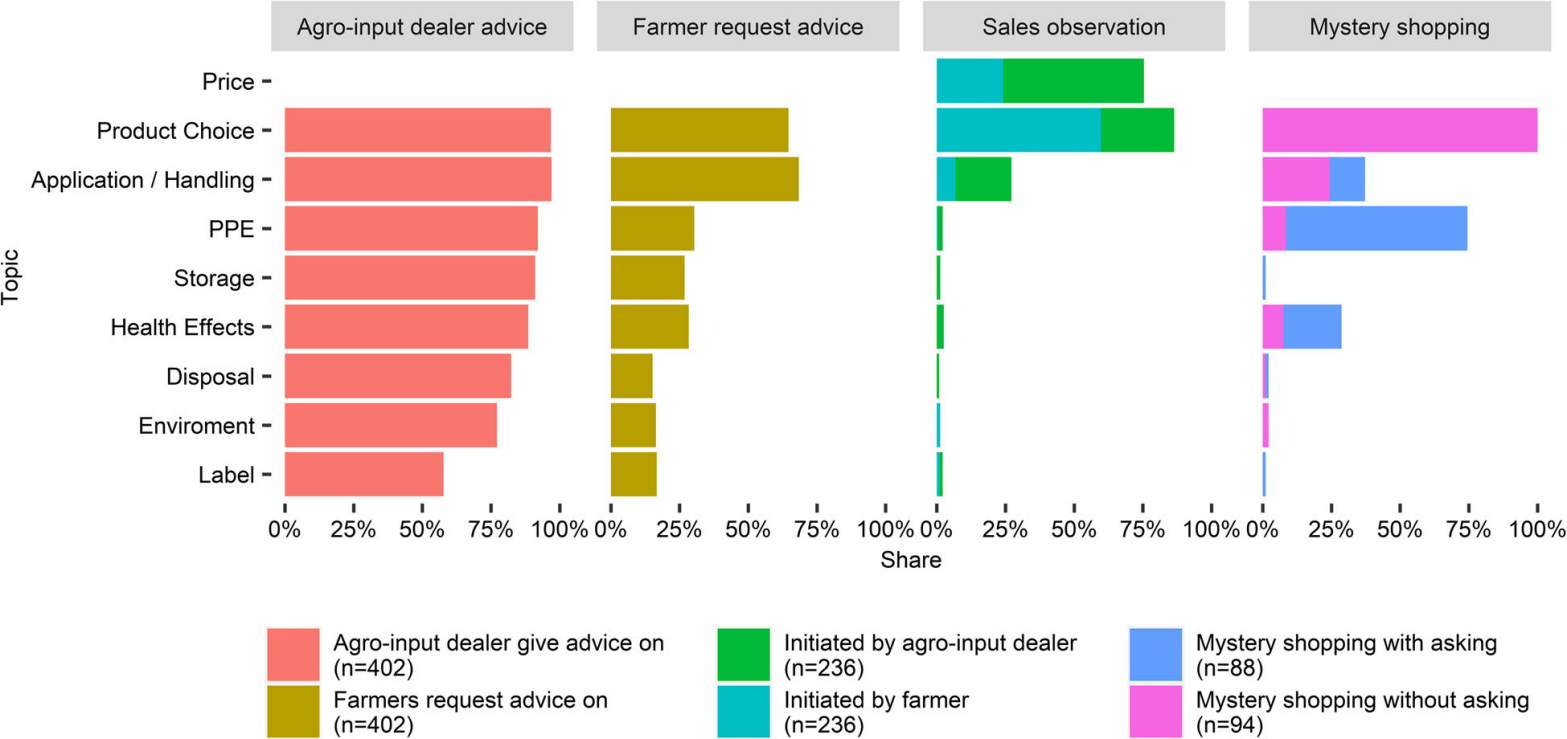
Comparison of mentioned topics in sale interaction by approach applied. Price as a topic was only investigated in sales observations. PPE: personal protective equipment. Reading example for 2nd column: 65% of agro-input dealers say that more than half their customers ask them to advise on product choice. * 100% Product choice in mystery shopping, due to the fact, that every farmer also purchased a product. Original questions for each section accessible in Supplementary Table ST 13, Additional File 1

The first column of Fig. 5 indicates that agro-input dealers self-reported that they commonly give advice on specific topics ranging from product choice and application and handling (both 97%) to label explanations (58%). The second column displays the share of agro-input dealers mentioning that 50% or more of farmers ask for advice on these topics. 65% of agro-input dealers claim their customers often ask for advice on product choice and 68% for advice on application and handling of pesticides. Sale observations (third column) revealed that product choice was indeed a topic in 86% of interactions and mostly initiated by the farmer, whereas dealers initiated conversations on price (a topic in 75% of interactions) and application and handling (28%). All other topics (such as use of PPE, adequate storage and disposal of pesticides, or health effects of pesticides) were rarely observed despite agro-input dealers claiming to give that type of advice regularly (Fig. 5, Column 1).

Lastly, the participatory observation during mystery shopping revealed that only twenty-seven shoppers (29%) were given any advice without asking for it and the topics were mainly application and handling (24%), followed by PPE (9%) and health effects (7%) (fourth column). No advice was given on safe storage or disposal of pesticides, nor about the impact on the environment. Product choice was discussed in all mystery shopping interactions, as the farmers were instructed to describe the problem but not ask for a specific product. The content of such a product choice discussion as well as discussions about the price were not further investigated.

After their pesticide purchase, the majority of the 94 mystery shoppers asked probing questions. Of the agro-input dealers who were asked the question “Is it dangerous for my health?”, the majority (79%) replied it was dangerous. To the follow-up question, “How should I protect myself?”, agro-input dealers primarily suggested the use of PPE (64%). However, the following quotes illustrate the range of answers and advice mystery shoppers were given when purchasing pesticides. For example, one agro-input dealer (female, 34, with certificate) answered that there is *“No side effect unless you drink it”.* Another agro-input dealer (female, 23, no certificate) advised *“After spraying you should also take some cold milk …*” . A third agro-input dealer (female, 37, no certificate) suggested to *“Look for other people to spray for you or just use it carefully”* as means of protection. In summary, farmers and agro-input dealers both focus on product choice and application procedures during sale interactions.

### Dealer knowledge

The question that arises from this lack of safe shops, proper pesticide sales, and needed advice on protective behavior for farmers is whether a lack of knowledge or attitudes is constraining agro-input dealers. First, all agro-input dealers were asked to identify and explain specific parts of an example pesticide hazard label (Fig. 3). Four out of five participants (79.9%) identified the colored label as indicating danger or hazards, with 15.2% naming it according to WHO guidelines ‘extremely dangerous’, while the others indicated statements such as (highly) hazardous, dangerous, (very) toxic, or fatal. Two out of five agro-input dealers were able to identify all other possible colors of pesticide labels (Supplementary Table ST 1, Additional File 1), while only one in eight agro-input dealers also correctly identified the corresponding meaning of these colors (Supplementary Table ST 14, Additional File 1). Only 43.3% of agro-input dealers identified all symbols on Fig. 3 correctly, while seven agro-input dealers (out of 402) identified none of the symbols. Wearing gloves was the symbol correctly identified by the most agro-input dealers (96.3%), while the least understood symbols were those related to the protection of other vulnerable life, such as children (36.6% wrong or no response), terrestrial (24.4%), or aquatic animals (22.1%) (Supplementary Fig. SF 4, Additional File 1).

Agro-input dealers were asked whether they understood the purposes of particular active ingredients (Table 3). Seven of the fifteen active ingredients scored below 50%. Carbaryl, carbofuran, and diazinon (all WHO toxicity class Ib or II) were least frequently correctly identified. Similarly, when asked for their best selling products, agro-input dealers reported brands, but were unaware of the corresponding active ingredients (Supplementary Table ST 15 and ST 16, Additional File 1). Moreover, around two out of five agro-input dealers were unable to name at least one pesticide banned in Uganda (active ingredients or corresponding brand; e.g. paraquat, DDT or carbofuran). In summary, knowledge on active ingredients is comparatively low.

**Table 3:** Identification of active ingredients vs. their use

Use identification (%)	Correct	Incorrect	Use unknown	AI unknown
2,4-D	96.0	1.2	2.0	0.8
Mancozeb	87.6	7.7	3.7	1.0
Cypermethrin	82.8	1.5	10.7	5.0
Glyphosate	82.6	0.8	10.7	6.0
Paraquat	70.4	4.5	19.2	6.0
Dimethoate	69.7	3.2	17.9	9.2
Profenofos	63.2	2.2	23.6	11.0
Diazinon	57.0	11.7	21.6	9.7
Carbofuran	48.5	11.9	27.6	11.9
Dichlorvos	44.8	2.2	32.6	20.4
Permethrin	34.8	3.2	40.3	21.6
Chlorpyrifos	33.3	3.2	36.8	26.6
Deltamethrin	29.4	2.5	43.3	24.9
Λ-Cyhalothrin	26.6	1.0	38.6	33.8
Carbaryl	10.0	3.5	52.2	34.3

*Note: The different columns denote correct (Yes) or incorrect (No) identification of the active ingredients’ (AI) use, knowing the AI name, but not its use (don’t know) or stating the AI is unknown. The proportion (last column) indicates the ratio between correct (yes) and wrong (no) answers*

*The fifteen AI were selected as most commonly used AI in the study area* [3]

### Dealer attitudes and beliefs

In addition to knowledge, agro-input dealers’ beliefs and feelings with regard to pesticides were investigated (Table 4). The agro-input dealers were provided with 30 statements with which they could agree or disagree (they were specifically told that these questions do not have a right or wrong answer). Fifteen out of thirty statements were agreed upon by less than 10% or more than 90% of agro-input dealers and revolved around topics of health and environmental risks, general protection, and farm profits. Nine statements were agreed upon by more than 30%, but less than 70% of agro-input dealers and revolved around pest management strategies (e.g. organic), pesticide effectiveness, and government oversight, but also safety aspects such as product labelling or product handling by customers.

**Table 4:** Questions investigating attitude towards pesticides. Sorted most to least agreement

Statement	yes (vs. no) %
Protective measures are necessary for pesticide use.	99.5
Pesticides contaminate water bodies.	97.8
You are worried about the toxicity of the chemicals to the people who use them or the people who eat the food.	97.3
You are worried about damaging the environment with toxic chemicals.	97.0
Pesticides can enter the body through the skin.	96.8
Pesticides can cause harm to the environment.	96.3
Using pesticides increases farm profits.	95.8
Pesticides affect livestock negatively.	94.3
You keep your pesticides inside the shop and out of reach of children and animals.	94.3
Pesticides have negative effects on the health of children.	93.3
When handling pesticides you are worried about getting cancer.	92.3
Pesticide use leads to soil degradation.	89.6
You are concerned about pesticide residues when buying vegetables from the market.	87.3
You think that the supply of agro-chemicals should be better controlled by the government.	86.1
Commercial production without pesticides is impossible.	78.6
Biopesticides are not as effective as chemical pesticides.	69.9
Organic agriculture is a good alternative to conventional agriculture.	69.7
You can determine whether a pesticide is dangerous or not by its smell.	53.5
Good pesticides are those that kill all insects immediately.	49.8
You think pesticide retailers are sufficiently monitored and supported by the government.	45.8
You think farmers apply the pesticides safely.	39.1
Mixing different pesticides makes the spraying more effective than using a single pesticide.	35.1
If there are many pests in the field then one should make the spraying mixture stronger.	34.6
You think colour codes on pesticides are not important.	30.6
Some pesticides have a pleasant smell.	26.4
Herbicides are not dangerous to humans.	23.4
Washing pesticide equipment in ponds or rivers does not affect the water quality.	9.7
Pesticides have a positive effect on beneficial species like bees or fish.	7.7
Empty pesticide containers can be reused for other purposes.	7.2
Drinking alcohol after spraying helps to eliminate side effects.	5.0

Original Text: “I would like to ask you some questions about your beliefs and feelings in relation to pesticides. When we say pesticides, we mean synthetic, chemical pesticides. There are no right or wrong answers in this section. We are interested in what comes to your mind immediately after hearing the statement. Please answer with either true or false only.” And “I would now like to ask you again some questions about your beliefs and feelings in relation to pesticides. Please answer this time with either yes or no.”

Additionally, agro-input dealers were asked questions around their self-perception as a source of information for farmers, as well as about their attitudes and beliefs with regard to the use of licenses, on counterfeits, pest resistance, and organic alternatives to synthetic pesticides. Almost all agro-input dealers (95.3%) perceived themselves to be a source of information to farmers, while just more than half (52.7%) of them thought that they were the best source of information for farmers in terms of safe pesticide use (Supplementary Fig. SF 5, Additional File 1). Almost nine out of ten agro-input dealers considered their shop license to be relevant. It enabled them to do business according to regulations (50.8%), enabled tax payment (20.7%), occupational safety (19.9%), and was a quality assurance to the customer (19.9%). Also, almost all agro-input dealers (93.3%) believed counterfeits were a big problem in Uganda and most believed (71.6%) they could identify a counterfeit. Seven out of ten (69.2%) had ever been concerned that the products they buy and sell could be counterfeits and three quarters (75.9%) of those had this worry in the last twelve months. Pest resistance was perceived to be a problem by 87.3% of agro-input dealers. The preferred strategies to address it were to better advise the farmer (33.8%) and to recommend stronger pesticides (23.6%) (Supplementary Table ST 17, Additional File 1). The majority (78.4%) of agro-input dealers was also aware of alternative approaches to chemical pest management, such as cultural, ecological, biological, and mechanical approaches (Supplementary Table ST 18, Additional File 1). However, agro-input dealers stated that alternatives are perceived as less effective and more time and labor consuming, although also cheaper, less skill-demanding, and with lower health risks. (Supplementary Fig. SF 6, Additional File 1). Most agro-input dealers stated that they recommend synthetic pesticides over alternatives (68.7%), mainly due to their effectiveness (90.5%) and economic benefits (92.5%). Those who recommended alternatives (31.3%), did so mainly to protect human health (80.0%) or the environment (78.1%) (Supplementary Table ST 19).

**Fig. 6:**
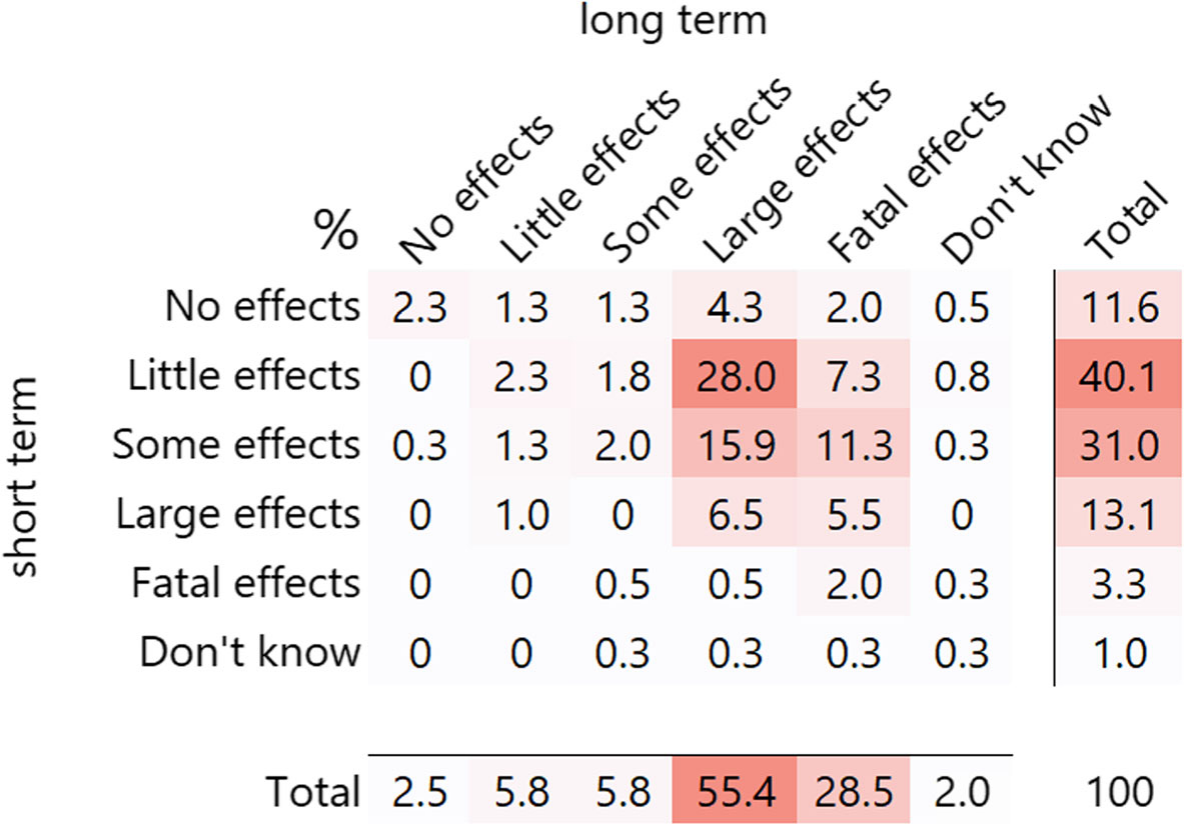
Assumed overall impact of pesticide handling and/or exposure on own short- and long-term health

Interestingly, almost all agro-input dealers believed that pesticides could affect their own health (98.8%). Most agro-input dealers assumed the short term effects to be *little* (39.9%), whereas the long-term effects were considered to be mostly *large* (54.7%) or *fatal* (28.1%) (Fig. 6). This was reflected in the terms agro-input dealers used for pesticide products when speaking with customers in their native language. The majority said they use the word for medicine (59.5%), followed by the word pesticide (30.1%) and lastly poison/toxin (9.5%).

Worryingly, more than two thirds (69.7%) of agro-input dealers had ever experienced health-related effects within 24 h after pesticide handling. The three most-recalled self-experienced symptoms were headache (29.1%), respiratory difficulties (23.6%), and skin irritation (22.4%). When asked about all possible symptoms of pesticide poisoning the most recalled were skin irritation (57.2%), headache (44.0%), itchy eyes (37.3%), and vomiting (33.3%) (Supplementary Table ST 20, Additional File 1). Close to half of all agro-input dealers (44.8%) recalled all four pesticide entry sites into the body (oral, dermal, inhalation, and ocular), whereas more than a quarter (28.9%) believed ears to be sites of entry (Supplementary Table ST 21, Additional File 1). Being aware of all possible entry sites is important as it can affect the PPE the agro-input dealer might recommend.

### Dealer outlook

The majority of agro-input dealers saw pesticide sales rise over the past five years (86.8%) and expect a further increase over the next five years (91.0%). The main explanations provided was an increase in farmers (31.1%) (Supplementary Table ST 22 and ST 23, Additional File 1). Agro-input dealers were also asked for their perspectives on possible future changes in the pesticide sector and all suggestions were agreed or strongly agreed upon by more than 80% of agro-input dealers (Supplementary Fig. SF 7, Additional File 1). The highest agreement was reached for reduced PPE pricing and an agro-input dealer certification of good practice. Furthermore, training needs to be decentralized and more affordable, as well as repeated even for established dealers. Organic farming demonstration plots, inputs suitable for organic farming, as well as a governmental strategy on organic farming are also desired. Least popular was the restriction and penalization of agro-input dealers who are not complying with regulations. Three in five (60.7%) agro-input dealers have a smartphone, while they estimate that on average only 24.6% of their customers have one. Two out of five agro-input dealers (40.3%) are subscribed to a text-message based service to receive regular messages with business-related information (Supplementary Table ST 24, Additional File 1).

### Regression

In a linear regression model (see Table 5), agro-input dealer knowledge significantly increases for every year increase in education (+ 0.115 points, *p* < 0.001), is higher in men (+ 0.577 points, p < 0.001), and in those with a certification of competency on safe handling of pesticide (+ 0.902 points, p < 0.001). On the other hand, agro-input dealer knowledge decreases for every 100 km distance from the capital Kampala (− 0.507 points, p < 0.001), after adjusting for all covariates. In contrast, agro-input dealer attitudes and beliefs become more focused on health and environment with increasing distance from the capital Kampala (+ 0.233, p < 0.001) and for each additional year of age (+ 0.018, *p* = 0.02), but less focused on these topics in those with a shop license (− 0.552 points, p = 0.02), after adjusting for all covariates. The number of safe pesticide handling and practices performed significantly decreases with increasing distance from Kampala (− 0.300, p < 0.001), is higher in men (+ 0.521, *p* = 0.008), and is higher in those with a certification of competency on safe handling of pesticide (+ 0.527, *p* = 0.01). Violations of laws and recommendations in the agro-input dealer shops are increasing with distance from Kampala (odds ratio: 1.65, p < 0.001). For the mystery shopping data the selected covariates were not significantly correlated with the toxicity of the recommended pesticide or advice giving. This could either be driven by an underpowered sample (*n* = 91) or shows that while agro-dealer have a higher knowledge about pesticides and report better practices with higher education and a certificate of competency and safe handling of pesticides, this does not translate into behavioral differences.

**Table 5:** Regression coefficients for the six dependent variables

Regression^a^	Knowledge			Attitudes & Beliefs			Practice			Laws &Recommendations			Toxicity			Advice given		
Coefficient^b^	Est	SE	*p*	Est	SE	*p*	Est	SE	*p*	OR	CI	*p*	OR	CI	*p*	OR	CI	*p*
Intercept	**3.057**	**0.449**	**0.000**	**10.92**	**0.438**	**0.000**	**7.570**	**0.608**	**0.000**	0.34	0.08; 1.47	0.150	11.00	0.32; 474.6	0.188	0.374	0.01; 9.18	0.545
Age (years)	0.003	0.008	0.710	**0.018**	**0.008**	**0.024**	0.006	0.011	0.585	0.97	0.89; 1.05	0.242	1.046	0.83; 1.32	0.172	1.027	0.84; 1.27	0.670
Education (years)	**0.115**	**0.026**	**0.000**	0.032	0.025	0.202	0.005	0.035	0.875	1.03	0.63; 1.68	0.460	0.705	0.18; 2.52	0.698	2.412	0.8; 8.01	0.795
Sex (male)	**0.577**	**0.144**	**0.000**	0.088	0.141	0.533	**0.521**	**0.195**	**0.008**	1.07	0.48; 2.25	0.820	2.147	0.46; 16.55	0.807	1.231	0.32; 4.25	0.794
Position (shop owner)	0.223	0.155	0.153	0.090	0.152	0.551	0.007	0.210	0.975	1.05	0.67; 1.67	0.743	1.173	0.33; 4.52	0.074	0.866	0.28; 2.52	0.422
CCSP^c^ (yes)	**0.903**	**0.152**	**0.000**	0.094	0.148	0.526	**0.527**	**0.206**	**0.011**	0.98	0.96; 1.01	0.906	0.959	0.9; 1.02	0.597	0.988	0.93; 1.04	0.129
Shop license (yes)	0.369	0.242	0.129	**−0.552**	**0.236**	**0.020**	0.184	0.328	0.574	1.09	0.66; 1.78	0.871	3.441	0.93; 14.6	0.384	0.638	0.21; 1.91	0.748
Distance to Kampala (100 km)	**−0.507**	**0.061**	**0.000**	**0.233**	**0.059**	**0.000**	**−0.300**	**0.082**	**0.000**	**1.65**	**1.35; 2.03**	**0.000**	1.039	0.61; 1.79	0.887	0.704	0.44; 1.1	0.130
Distance to next city (100 km)	−0.179	0.264	0.498	0.207	0.257	0.421	0.532	0.357	0.137	1.54	0.67; 3.6	0.310	0.216	0.01; 2.52	0.245	2.276	0.32; 18.04	0.416
Data^d^	KAP			KAP			KAP			KAP			MYS			MYS		
Observations (n)	397			397			397			397			91			91		
Adjusted *r*^*2* e^	0.337			0.065			0.072			NA			NA			NA		
Score range	0–10			0–16			0–12			0 / 1			0 / 1			0 / 1		
Distribution	Normal			Normal			Normal			Binomial			Binomial			Binomial		

*Notes: bold coefficients denote a p-value below 0.05*

^*a*^*The six dependent variables were ‘pesticide knowledge for label, use and alternatives’, ‘attitudes and beliefs towards the safe pesticide use’, ‘safe pesticide handling and practices’, ‘following the laws and recommendations in the shop setup and organization’, ‘toxicity of sold products’, ‘advice given during mystery shopping’*

^*b*^*Regression coefficients are abbreviated for estimate (Est), standard error (SE), odds ratio (OR), 95% confidence interval (CI) and p-value (p)*

^*c*^*CCSP: Certification of competency on safe handling of pesticide*

^*d*^*Datasets used for the regression were either from the interview of agro-input dealers (KAP) or from the mystery shopping (MYS)*

^*e*^*r*^*2*^*is not available (NA) for the three binomial regressions*

## Discussion

This study applied three different approaches to illustrate agro-input shop conditions and products available, agro-input dealers’ pesticide advice for smallholder famers, and agro-input dealers’ knowledge, attitude and practices in terms of pesticides and the related risks to human and environmental health. The findings display a gap between stated and observed behavior in advising customers, suggesting important opportunities for dealer professionalization and improvement of risk communication towards smallholder farmers.

The findings demonstrate that 97% of agro-input dealers perceive it as their responsibility to advise farmers, which is an increase of 13% from the results presented in the 2009 census [27]. While the majority of agro-input dealers claims to advise farmers on health and environmental effects, storage, disposal, PPE, and labels, observation of sales interactions revealed that, with rare exception, product choice, price, and application practices are the only topics discussed. Farmers are not asking for topics beyond these and agro-input dealers hence do not share further information they might have with the farmer. Mystery shopping has shown that when asked, agro-input dealers can also advise smallholders on health topics, but without necessarily providing best practice answers. Although we know that awareness does not always translate into action [1–3], it is still essential that farmers are informed about health and environmental risks as well as their prevention. While regulators and the WHO consider the label one of the main tools to share risk, safety, and health information on pesticides, the evaluation in this study of agro-input dealers’ advice has shown that label explanation is rare. Previous research showed that label information does not reach farmers when they are unaware of its importance. If agro-input dealers explained the label more frequently, they could help farmers overcome hurdles in literacy, language, and access to labels [11].

An explanation for the absence of risk-advice giving practices could be the domination of the pesticide-value chain by immediate profit motives, which has been suggested in Ethiopia [17]. The combination of a knowledge monopoly in the last mile [44] and the absence of a competitive advantage for environmental and health advice places the smallholder farmer in a vulnerable position, with no other access to this information. This effect is amplified in low productivity areas, where the farmers are also underserved in health care. In these areas, an untreated pesticide poisoning could result in larger health effects, such as permanent neurological damage or reproductive effects [45].

A second possible explanation for the gap between perceived responsibility and advice given is the lack of appropriate training. The findings have shown that while the majority of agro-input dealers fulfill the criteria for general education, specialized training provided through the certification of competency on safe handling of pesticide was only attained by about half the interviewed agro-input dealers (55.7%). This is in line with previous studies from low- and middle income countries claiming agro-input dealers are lacking education and training, therefore giving smallholders access to hazardous chemicals without appropriate stewardship [14, 15]. The regression analysis shows that a certificate increases the practice and knowledge score, while years of education alone only increases the knowledge score. This underlines the importance of the certification course, which is currently offered at Makerere University in the capital Kampala. Travelling to Kampala and staying in town is expensive and time consuming for those far from the capital. Distance from Kampala, where the pesticide certification course is offered, is significantly associated with lower pesticide knowledge, attitude and practices, as well as lower compliance with laws and recommendations. This suggests that travel distance may be a major barrier to accessing training on pesticide use and handling, and supports the development of more decentralized training programs. After certification, the agro-input dealers also have to undergo a long and expensive process to register their businesses. Together, this may be too expensive for new businesses, crippling them before they have established viability.

Most agro-input dealers agree that the trainings need to be decentralized and more affordable. A subsidized collaboration with pesticide suppliers as well as specialists for environment and health could tour different cities, thereby eliminating agro-input dealers’ need to travel far from their business. These traveling trainings could focus on ensuring that agro-dealers hear not only about the economic benefits of pesticides, but also the risks. The present study indicates that knowledge retention is not yet ideal, suggesting repetition courses would be a useful tool to avoid knowledge loss over time. Furthermore, two studies from Nepal have shown that agro-input dealer training significantly increased their knowledge on pesticide hazards and reduced sales of unregistered pesticides [46], but agro-input dealers lacked the incentives to adopt other necessary safety measures in pesticide handling, thus missing the opportunity to be a role-model to their customers [47].

Likewise, this study showed that not all agro-input dealers are a good example for farmers when it comes to personal protection and hygiene during pesticide handling. Precautionary practices, such as avoiding eating and drinking, regular handwashing, and the use of PPE are not trivial if a shop is open 12 h per day, every day of the week, while tap water is lacking and PPE inaccessible. Furthermore, even where the conditions are ideal, these practices can be uncomfortable or agro-input dealers can believe they are not needed. This lack of precautionary practices by agro-dealers contrasts with the belief of the vast majority of agro-input dealers that pesticides can affect their health (98.8%). This belief seems to stem from their own experiences, such as self-reported symptoms of intoxication, their experience with farmers having pesticide poisonings, or even use for self-harm. Nevertheless, when asked to categorize health effects into short and long term, it becomes evident that the downsides of pesticide use are mentally postponed into a distant future, where they are all the more harmful and costly [48]. This is in line with the concept of discounting, where the benefits of today are valued higher than the losses of the future [49]. When targeting agro-input dealers for a behavior change intervention, this context needs to be taken into account.

A third explanation as to why risk-advice is missing could be that the curriculum for the certification of competency on safe handling of pesticide may be targeted only at what agro-input dealers should know and not at how agro-input dealers should transfer their knowledge to the farmer. An approach to standardize the sale interaction between smallholder farmers and agro-input dealers would be to train them similarly to pharmacists [50]. In this model, agro-input dealers would always explicitly ask the customer whether they need information on pesticide storage, container disposal, PPE, and the like. Logistically, the number of customers per shop indicates that training agro-input dealers in risk communication would reach more than one thousand farmers per season per shop. An alternative lever would be the use of free smartphone applications advising on pest management practices and related safety measures. In this survey, 61% of agro-input dealers and around 25% of their customers already have a smartphone. A study researching the effects of text message services on the behavior of their subscribers (40% of agro-input dealers in this survey) could provide insights into how to shift agro-input dealers towards new business models based on services instead of or additional to product sales.

Service provision could also resolve the economic threat agro-input dealers see in pest management practices involving reduced pesticide use. Pesticides are currently the best-selling and most profitable products of almost all agro-input dealers. This research reveals that many agro-input dealers are ready to shift away from this model and instead focus on guiding farmers on different pest management practices, including organic. Attitudes revealed that those agro-input dealers who recommended alternatives to synthetic products (31.3%) did so to protect human and environmental health. While agro-input dealers perceive these alternatives to be more time consuming and labor intense they are also deemed cheaper and less skill-demanding. This is also supported by the majority of agro-input dealers agreeing that Uganda needs a national strategy on organic farming as well as more products suitable for organic farming.

Fourth, similar to the 2009 census, most agro-input shops have only been in business for a few years and the employees’ experience in selling pesticides was even shorter. Furthermore, 90% of the interviewed agro-input dealers saw an increase in pesticide sales over the last five years and expect a further increase for the next five. This could be a sign of a rapidly increasing market, emphasizing the need for proper guidance and training of agro-input dealers, as well as an aggravation of the current situation in the future. It also raises the question of whether the staff in these new shops possess adequate knowledge and experience to encourage farmers to reach out to them for advice. The relative number of female agro-dealers is surprisingly high and in contrast to previous studies [15, 19, 22, 27]. This may reflect a recent trend for increasing employment of women in the agro-industry in Uganda, or may be the result of our unannounced visits.

The most sold products in the mystery shopping and as self-reported by agro-input dealers are WHO toxicity classes Ib or II, indicating a moderate or high acute toxicity for adult humans. In our specific mystery shopping example, the list of recommended products against the fall armyworm included five of 13 products in toxicity class III and U. This indicates that in addition to not advising customers on the risks, agro-input dealers also do not prioritize products of lower toxicity. In the mystery shopping, purchased products other than class II were somewhat more expensive. A possible explanation for higher prices for class III and U could be that these products are often more specific, thus less in-demand. Another explanation may be that agro-input dealers perceive broad-spectrum pesticides (which are most often also the cause of the higher toxicity) as more effective or are more experienced with them. When comparing the WHO toxicity classes found in previous research, there seems to be no change away from the products of class I and II [1–3], indicating an explicit choice by agro-input dealers to keep these products on the market. This means that a change is currently unlikely, indicating the need for actors higher up in the value-chain to place more emphasis on the risks of such pesticides.

Upstream pesticide value-chain-actors can use different mechanisms to steer farmers towards specific products. In Switzerland, for example, a study has shown that farmers advised by public extension were more likely to use preventive measures, while farmers advised by private extension were more likely to use synthetic insecticides [51]. In our study, mystery shopping has revealed that not all agro-input dealers follow the recommendations of the Ministry of Agriculture, Animal Industry and Fisheries. It is unclear, however, what criteria the Ministry applied to select the recommended products and how they communicated this to the agro-input dealers. Similarly, most agro-input dealer shops were not registered with the Ministry or were never or only initially inspected. All shops show minor deviations from recommended practices, while a third of shops show very serious deviations (e.g. repackaged or unmarked containers, food on sale). Previously, governmental regulating bodies, as well as Uganda National Agro-Input Dealer Association, have expressed the need for governmental inspection of their shops, to prevent and control such deviations in a timely manner [52]. Such inspections would follow environmental governance standards advocating for inclusion of those directly responsible for a problem in also governing it [53].

### Strengths and limitations

This study is the first among agro-input dealers in low- and middle-income countries using a mixed-methods approach to collect data on both stated and observed behavior of agro-input dealers to describe their pesticide sales and information behavior towards farmers as well as the knowledge, attitude, and pesticide handling practices in their shops. The combination of approaches allowed us to account for social desirability- and recall bias, leading to more accurate results. Moreover, our approach of taking a random selection of agro-input dealers from a given sampling frame allows us to extrapolate data to other agro-input dealers working under similar conditions in similar cultural, economic, and agricultural circumstances.

A possible bias may have been introduced through the data collection method and process, as the number of agro-input dealers visited over a brief period was high and the survey comparatively long, which may have left agro-input dealers or interviewers tired and thus answering or collecting data inaccurately. However, neither inquiries with the interviewers nor the low number of incomplete interviews support this hypothesis. Furthermore, when comparing interview data to the observational findings, we need to be aware that the self-reported interview data represent an average perspective from the agro input-dealer, whereas the observation is a one-time situational assessment, therefore not accounting for intra-personal or temporal-variability.

For the mystery shopping it was critical that the investigated employees believed they were interacting with a real customer. Local farmers were recruited and systematically trained for their role as mystery shoppers. In order to have comparable mystery shopping data, the farmers always presented the same problem to the agro-input dealers. The self-reported data from mystery shoppers was systematically retrieved during the debriefing. While none of the data support this conclusion, it is still possible that recruiting different farmers for different observations may have introduced variance in the mystery shopping experience reporting.

While we did analyze if agro-input dealers gave advice on a certain topic, we did not systematically analyze the content of the advice given and whether it was correct. Moreover, we did not assess the long-term outcome of whether there is a connection between the lack of advice and farmers’ handling of pesticides, resulting in negative health and environmental impacts. Despite these limitations, we believe that due to the low number of studies on agro-input dealers’ knowledge, attitude and practices, our research remains highly insightful.

## Conclusion

This research among 402 Ugandan agro-input dealers is the first to systematically collect data on both stated and observed behavior towards farmers. Combined with the collected information on agro-input dealers’ knowledge and agro-input dealers’ shops, it provides useful and novel insights. Training, certification, registration, and licensing, a combination of efforts to ensure the health and safety of the agro-input dealers in their shops, their customers, their environment and communities are underway, but far from complete. With the rapid increase in pesticide use, it is imperative to make agro-input dealer training accessible and affordable and specifically targeted at providing a comprehensive service to farmers. Shifting agro-input dealers’ business model away from product sales and more towards service provision could reduce conflicting economic incentives to sell as many products as quickly as possible. Governmental and private actors should streamline the pesticide value chain to provide equitable access to appropriate tools and information, avoiding a worsening of the status quo in the future.

## Supplementary Information

**Additional file 1: Table ST 1.***.* WHO toxicity classes and hazard color band. **Table ST 2.** Label explanation. **Table ST 3.** Highest Qualification to be an agro-input dealer. **Table ST 4.** Content of general pesticide training. **Table ST 5.** Training providers for general pesticide training as well as specific training on pesticide alternatives and pesticide application. **Table ST 6.** Inspection and License. **Table ST 7.** Categorization of deviations from recommended shop organization and setup. **Table ST 8.** Hygiene practices. **Table ST 9.** Container handling practices and disposal. **Table ST 10.** Stocked products, their availability, bestsellers, profitability and future offerings. **Table ST 11.** PPE available for sale. **Table ST 12.** Suggested and purchased products during mystery shopping. **Table ST 13.** Original questions to Fig. 5. **Table ST 14.** Label colors and areas. **Table ST 15.** Brands mentioned as best, second or third selling product. **Table ST 16.** Corresponding active ingredients to best, second or third selling product. **Table ST 17.** Agro-input dealers’ attitudes regarding license, counterfeits and management of pest resistance. **Table ST 18.** Alternatives to synthetic pesticides and their limitations. **Table ST 19.** Recommendations and corresponding reasons. **Table ST 20.** Symptoms of pesticide poisoning recalled (known) or experienced. **Table ST 21.** Through which body parts do you think pesticides can enter us?. **Table ST 22.** Pesticide trends over the past and future five years within community. **Table ST 23.** Reasons for pesticide trends*.* **Table ST 24.** What companies are you subscribed to receive regular messages with business-related information on your mobile phone?. **Table ST 25.** Detailed safety equipment layout. **Fig. SF 1.** PPE access and use for agro-input dealers when handling pesticides. **Fig. SF 2.** Availability of pesticides in shops by WHO toxicity class. **Fig. SF 3.** Approved pesticides available for controlling the fall armyworm in Uganda. **Fig. SF 4.** Hazard symbol identification. **Fig. SF 5.** Information sources of farmers according to agro-input dealers. **Fig. SF 6.** Comparison of synthetic pesticides with alternatives to them. **Fig. SF 7.** Attitudes regarding future possible change in the pesticide sector in Uganda.

## Data Availability

The datasets used and/or analyzed during the current study are available from the corresponding author on reasonable request. The datasets generated and/or analyzed during the current study are available in the Eawag research data institutional repository (ERIC): 10.25678/0004CX.
