# Supplementary material for: What agro-input dealers know, sell and say to smallholder farmers about pesticides: a mystery shopping and KAP analysis in Uganda
Source: Environ Health. 2021 Sep 1;20:100. doi: 10.1186/s12940-021-00775-2 (PMC8411546; doi:10.1186/s12940-021-00775-2)
Supplement: Supplementary file 1 — Additional file 1: Table ST 1.. WHO toxicity classes and hazard color band. Table ST 2. Label explanation. Table ST 3. Highest Qualification to be an agro-input dealer. Table ST 4. Content of general pesticide training. Table ST 5. Training providers for general pesticide training as well as specific training on pesticide alternatives and pesticide application. Table ST 6. Inspection and License. Table ST 7. Categorization of deviations from recommended shop organization and setup. Table ST 8. Hygiene practices. Table ST 9. Container handling practices and disposal. Table ST 10. Stocked products, their availability, bestsellers, profitability and future offerings. Table ST 11. PPE available for sale. Table ST 12. Suggested and purchased products during mystery shopping. Table ST 13. Original questions to Fig. 5. Table ST 14. Label colors and areas. Table ST 15. Brands mentioned as best, second or third selling product. Table ST 16. Corresponding active ingredients to best, second or third selling product. Table ST 17. Agro-input dealers’ attitudes regarding license, counterfeits and management of pest resistance. Table ST 18. Alternatives to synthetic pesticides and their limitations. Table ST 19. Recommendations and corresponding reasons. Table ST 20. Symptoms of pesticide poisoning recalled (known) or experienced. Table ST 21. Through which body parts do you think pesticides can enter us?. Table ST 22. Pesticide trends over the past and future five years within community. Table ST 23. Reasons for pesticide trends. Table ST 24. What companies are you subscribed to receive regular messages with business-related information on your mobile phone?. Table ST 25. Detailed safety equipment layout. Fig. SF 1. PPE access and use for agro-input dealers when handling pesticides. Fig. SF 2. Availability of pesticides in shops by WHO toxicity class. Fig. SF 3. Approved pesticides available for controlling the fall armyworm in Uganda. Fig. SF 4. Hazard symbol identification. Fig [file 12940_2021_775_MOESM1_ESM.docx]

# Additional File 1 to Staudacher, Brugger et al. 2021 - What agro-input dealers know, sell and say to smallholder farmers about pesticides: A mystery shopping and KAP analysis in Uganda.

Supplementary Table ST 1: WHO toxicity classes and hazard color band. Adapted from WHO (42) and FAO and WHO (9). LD_50_: Lethal dose whereby 50% of the animals die

| WHO Toxicity Class | | LD_50_ for rat (mg/kg body weight) | |
| --- | --- | --- | --- |
| Class* | **label** | **Oral** | **Dermal** |
| Ia | Extremely hazardous | < 5 | < 50 |
| Ib | Highly hazardous | 5 – 50 | 50 – 200 |
| II | Moderately hazardous | 50 – 2000 | 200 – 2000 |
| III | Slightly hazardous | > 2000 | > 2000 |
| IV / U | Unlikely to present acute hazard | > 5000 | > 5000 |

Supplementary Table ST 2: Label explanation

| Part of Label | Explanation |
| --- | --- |
| Symbol 1 | Keep locked away and out of reach of children |
| Symbol 2 | Wear rubber boots |
| Symbol between 2 and 3 | Wear rubber apron |
| Symbol 3 | Wear overalls |
| Symbol 4 | Wear gloves |
| Symbol between 4 and (4) left | Handling of product |
| Symbol between 4 and (4) right | Application of product |
| Symbol between (4) and 5 | Wear mask with carbon filter |
| Symbol 5 | Dangerous/harmful to animals |
| Symbol 6 | Dangerous/harmful to fish – do not contaminate lakes, rivers, ponds or streams |
| Symbol 7 | Wear eye protection |
| Symbol 8 | Wash after use |
| Reading from left to right | Order of actions to be conducted |
| Warning color red | WHO toxicity class Ia/Ib |

Supplementary Table ST 3: Highest Qualification to be an agro-input dealer

| Highest Qualification to be an agro-input dealer | Unit | KAP^a^ | OBS^a^ | MYS^a^ |
| --- | --- | --- | --- | --- |
| Degree AVPM^b^ | % | 5.2 | 5.9 | 6.4 |
| Diploma in AVPM^b^ | % | 10.7 | 11.4 | 10.6 |
| Certificate in AVPM^b^ | % | 13.4 | 13.1 | 11.7 |
| Deg. /Dip. /Cert. in Business, Admin., Accounting, etc. | % | 19.7 | 21.6 | 20.2 |
| Advanced secondary (A Level) without additional training | % | 9.7 | 10.2 | 12.8 |
| Ordinary secondary (O Level) without additional training | % | 24.6 | 22.5 | 27.7 |
| Below O Level without additional training | % | 16.7 | 15.3 | 10.6 |

Note: No significant differences were found.
^a^The samples are abbreviated with KAP for the full sample of interviewees, MYS for those participating in Mystery Shopping and OBS for those participating in the sales observation
^b^AVPM: Agriculture, Veterinary, Pharmacy or Medicine

Supplementary Table ST 4: Content of general pesticide training

| Topic | % |
| --- | --- |
| Safe use and handling of chemicals (or pesticides) | 86.9 |
| (New) product knowledge | 32.9 |
| Crop protection (Pest and disease identification & product matching) | 20.8 |
| General agriculture | 18.5 |
| Business management | 23.0 |
| Don't know / No response | 2.6 |

Supplementary Table ST 5: Training providers for general pesticide training as well as specific training on pesticide alternatives and pesticide application. MAAIF: Ministry of Agriculture, Animal Industry and Fisheries.

|  | General Training (%) | | Alternatives (%) | | Application (%) | |
| --- | --- | --- | --- | --- | --- | --- |
| Base for share (number) | n=402 | n=313 | n=402 | n=176 | n=402 | n=363 |
| Ever attended a training on pesticides … | 77.9 | 100.0 | 43.8 | 100.0 | 90.3 | 100.0 |
| Informal training from shop owner | 16.4 | 21.1 | 5.2 | 11.9 | 20.4 | 22.6 |
| MAAIF or other national government agency | 16.7 | 21.4 | 6.0 | 13.6 | 20.1 | 22.3 |
| Pesticide manufacturer, importer or supplier | 4.5 | 5.8 | 2.0 | 4.5 | 8.0 | 8.8 |
| Local government, such as agricultural extension | 7.0 | 8.9 | 4.5 | 10.2 | 7.7 | 8.5 |
| Schools or university | 14.7 | 18.8 | 16.7 | 38.1 | 27.4 | 30.3 |
| UNACOH (Uganda National Association for Community and Occupational Heath) | 1.0 | 1.3 | 0.2 | 0.6 | 1.0 | 1.1 |
| UNADA (Uganda National Agro Input Dealer Association) | 18.4 | 23.6 | 7.7 | 17.6 | 21.1 | 23.4 |
| Crop Life (Umbrella Pesticide Importer Association) | 2.5 | 3.2 | 0.7 | 1.7 | 0.7 | 0.8 |
| NOGAMU (National Organic Agricultural Movement of Uganda) | 0.5 | 0.6 | 0.0 | 0.0 | 0.5 | 0.6 |
| Media (radio / TV / newspaper) | 3.2 | 4.2 | 2.7 | 6.3 | 2.7 | 3.0 |
| Self-trained through product labels or supplier leaflets | 3.0 | 3.8 | 2.7 | 6.3 | 8.0 | 8.8 |
| NGO | 2.0 | 2.6 | 1.2 | 2.8 | 1.2 | 1.4 |
| Agribusiness | 5.5 | 7.0 | 1.0 | 2.3 | 2.5 | 2.8 |
| USAID / Feed the Future | 2.2 | 2.9 | 0.2 | 0.6 | 1.5 | 1.7 |
| Fellow Farmers / Cultural Practice | 0.0 | 0.0 | 3.2 | 7.4 | 1.0 | 1.1 |
| Other | 0.2 | 0.3 | 0.0 | 0.0 | 0.0 | 0.0 |
| Don't remember | 5.5 | 7.0 | 2.5 | 5.7 | 2.7 | 3.0 |
| No response | 0.0 | 0.0 | 0.0 | 0.0 | 0.2 | 0.3 |

Supplementary Table ST 6: Inspection and License. MAAIF: Ministry of Agriculture, Animal Industry and Fisheries

| Has your shop ever been inspected by an authority, and what for? | % |
| --- | --- |
| No inspection | 16.2 |
| initial license approval or license renewal | 30.6 |
| Quality control: Counterfeits, fake, unregistered, unauthorized, outdated products | 36.8 |
| inspection of the shop/setup | 10.2 |
| sensitization | 3.0 |
| other | 0.5 |
| Don't Know | 1.0 |
| No response | 1.7 |
| **Is the shop licensed as pesticide distribution store with MAAIF** | **%** |
| No | 41.5 |
| In progress | 17.7 |
| Yes without evidence | 23.9 |
| Yes with evidence: license not up-to-date | 2.74 |
| Yes with evidence: license up-to-date | 5.72 |
| Don't Know | 7.96 |
| No response | 0.5 |

Supplementary Table ST 7: Categorization of deviations from recommended shop organization and setup.

| Deviation | somewhat serious | serious | very serious |
| --- | --- | --- | --- |
| **Documents** | **85.7%** |  |  |
| Display of CCSP | 58.4% No |  |  |
| Display of business license | 71.7% No |  |  |
| Product records | 38% No |  |  |
| **Shop organization** | **20.2%** | **25.5%** | **7.7%** |
| Clean and orderly shop | 20.2% No |  |  |
| Food on sale in shop |  |  | 6.6% Yes |
| Animal feed on sale in shop |  |  | 1.3% Yes |
| Neighboring shops selling food or animal feed |  | 25.5% Yes |  |
| **Containers** | **90.3%** |  | **30.6%** |
| (Restricted) pesticides under lock | 90.3% No |  |  |
| Unmarked/unlabeled containers |  |  | 10.5% Yes |
| Repackaged containers |  |  | 25% Yes |
| Leaking containers |  |  | 6.1% Yes |
| **Displays** | **99.7%** |  |  |
| Displaying general health and safety information | 87.2% No |  |  |
| Displaying warnings on pesticides | 94.9% No |  |  |
| Displaying prohibition of smoking, eating and drinking | 93.4% No |  |  |
| Displaying prohibition of underage pesticide sales | 99% No |  |  |
| **Infrastructure** | **99.7%** | **89.8%** | **2.8%** |
| Shop size > 9m2 |  | 41.1% No |  |
| Shelves for pesticide storage | 25.5% > 2.5m | 3.6% No |  |
| Palettes for pesticide storage | 6.1% > 1.3m | 41.6% No |  |
| Pesticide exposure to sunlight, water or moisture |  | 7.7% Yes |  |
| Pesticides stored separately from other commodities | 20.9% No |  |  |
| Shop walls from washable materials | 23.2% No |  |  |
| Shop floor from washable materials | 18.4% No |  |  |
| Shop floor drainage | 78.8% No |  |  |
| Sufficient lighting | 6.1% No |  |  |
| Sufficient ventilation |  | 31.1% No |  |
| Sufficient water supply |  | 43.4% No |  |
| Electric wires in wall tubes | 42.9% No |  |  |
| Fire Fighting equipment | 93.4% No |  |  |
| Unobstructed fire exit |  | 41.6% No |  |
| Lockable doors |  |  | 2.8% No |
| **Safety Equipment*** |  | **90.1%** |  |
| No PPE visible |  | 61.2% Yes |  |
| Nothing to wash eyes or remove toxic materials visible |  | 41% Yes |  |
| Soap and water (tap/bucket) visible |  | 75.5% No |  |
| No materials for cleanup or disposal visible |  | 41.8% Yes |  |
| Broom visible |  | 43.4% No |  |
| **Total** | **100%** | **98%** | **36%** |

*Safety Equipment is categorized based on subsets of questions given in Supplementary Table ST 25
CCSP: Certification of competency on safe handling of pesticide

Supplementary Figure SF 1: PPE access and use for agro-input dealers when handling pesticides.

Supplementary Table ST 8 Hygiene practices

| How long after you handled pesticides do you take a bath? | % |
| --- | --- |
| Immediately after | 22.64 |
| A few hours later | 9.45 |
| Many hours later | 64.43 |
| The next day or later | 1.24 |
| Not applicable | 0.75 |
| No response | 1.49 |
|  |  |
| **How long after you handled pesticides do you change your clothes?** | **%** |
| Immediately after | 16.92 |
| A few hours later | 14.68 |
| Many hours later | 63.43 |
| The next day or later | 2.24 |
| Not applicable | 1.49 |
| No response | 1.24 |
|  |  |
| **Who washes the clothes you wore during pesticide handling?** | **%** |
| Me | 66.67 |
| A family member | 23.38 |
| Maintenance aid or washerwoman of the shop | 7.46 |
| They aren't washed | 0 |
| No response / Don't know / etc. | 2.49 |

A minority (8.5%) had refillable containers in stock, but nineteen out of twenty (94.8%) of agro-input dealers said none of the farmers ever returned containers to them.

Supplementary Table ST 9: Container handling practices and disposal

| Why have you stopped repackaging or mixing pesticides in your shop? | % |
| --- | --- |
| health effects | 33.33 |
| personal health effects | 20.51 |
| it's illegal | 28.21 |
| packaging changed | 7.69 |
| Other | 5.13 |
| No response | 5.13 |
|  |  |
| **How are you disposing of empty pesticide containers?** | **%** |
| I don't dispose of any empty containers | 45.0 |
| Municipal disposal site / waste / trash | 11.7 |
| Burning | 36.3 |
| Burying | 5.5 |
| Recycling to manufacturer | 2.2 |
| Reused for pesticide refill | 0.7 |
| Reused for other purposes | 2.0 |
| Other | 0.5 |
| Don't know | 0.5 |
| No response | 0.5 |
|  |  |
| **How are you disposing of waste pesticides?** | **%** |
| There are no waste pesticides | 33.1 |
| Municipal disposal site / Waste / Trash | 19.7 |
| Burning | 12.9 |
| Burying | 8.7 |
| Recycling to manufacturer | 24.1 |
| They are sold to customers | 1.0 |
| Apply in own garden | 4.5 |
| Other | 1.0 |
| Don't know | 0.7 |
| No response | 0.5 |

Note: Waste pesticides are pesticides that have expired or are excess pesticides and need to be disposed of.

Supplementary Table ST 10: Stocked products, their availability, bestsellers, profitability and future offerings

| Products (n=402, %) | available | most sold | most profitable | offered in the future |
| --- | --- | --- | --- | --- |
| Herbicides (synthetic) | 97.5 | 47.3 | 50.7 | 4.2 |
| Insecticides (synthetic) | 95.3 | 33.3 | 22.9 | 4.0 |
| Fungicides (synthetic) | 87.3 | 8.0 | 6.7 | 1.5 |
| Rodenticides | 30.3 | 0.2 | 0.2 | 0.0 |
| Nematicides | 14.2 | 0.7 | 0.5 | 0.7 |
| Acaricides | 4.5 | 0.0 | 0.0 | 0.2 |
| Organic pesticides | 10.4 | 1.5 | 0.5 | 1.0 |
| Insect pheromones | 4.0 | 0.2 | 0.0 | 0.2 |
| Veterinary products besides acaricides | 2.5 | 0.2 | 0.2 | 0.2 |
| Fertilizer | 92.3 | 2.5 | 6.0 | 2.7 |
| Seeds | 85.6 | 2.0 | 5.7 | 4.7 |
| Spray Pump | 65.4 | 0.0 | 0.7 | 2.0 |
| Farm Tools and Equipment | 42.5 | 0.2 | 0.2 | 11.7 |
| PPE | 48.8 | 0.2 | 0.5 | 13.4 |
| Processing and Packaging Equipment | 3.2 | 0.0 | 0.0 | 0.5 |
| Animal Feed | 1.2 | 0.2 | 0.0 | 0.7 |
| Food | 0.7 | 0.0 | 0.0 | 0.0 |
| Hygiene articles | 0.2 | 0.0 | 0.0 | 0.5 |
| Human medicine | 0.2 | 0.0 | 0.0 | 0.0 |
| Spray Pump spares | 3.7 | 0.0 | 0.0 | 0.0 |
| Other | 2.2 | 0.7 | 0.2 | 0.7 |
| Don't Know | 0.5 | 0.2 | 2.0 | 33.3 |
| No response | 1.5 | 2.2 | 2.7 | 17.4 |

Supplementary Table ST 11: PPE available for sale

|  | Share of shops (%) | Share of shops offering PPE (%) |
| --- | --- | --- |
| Base for share (number) | n=402 | n=196 |
| Cap | 1.2 | 2.6 |
| Glasses | 11.7 | 24.0 |
| Mask with carbon filter | 17.4 | 35.7 |
| Mask without carbon filter | 31.6 | 64.8 |
| Long sleeved shirt | 0.5 | 1.0 |
| Poncho | 0.0 | 0.0 |
| Overall or kimono | 3.0 | 6.1 |
| Rubber apron | 0.2 | 0.5 |
| Gloves | 27.6 | 56.6 |
| Long pants | 0.2 | 0.5 |
| Waterproof pants | 0.5 | 1.0 |
| Gaiters | 0.2 | 0.5 |
| Gumboots | 35.6 | 73.0 |
| Other | 0.2 | 0.5 |

Supplementary Figure SF 2: Availability of pesticides in shops by WHO toxicity class


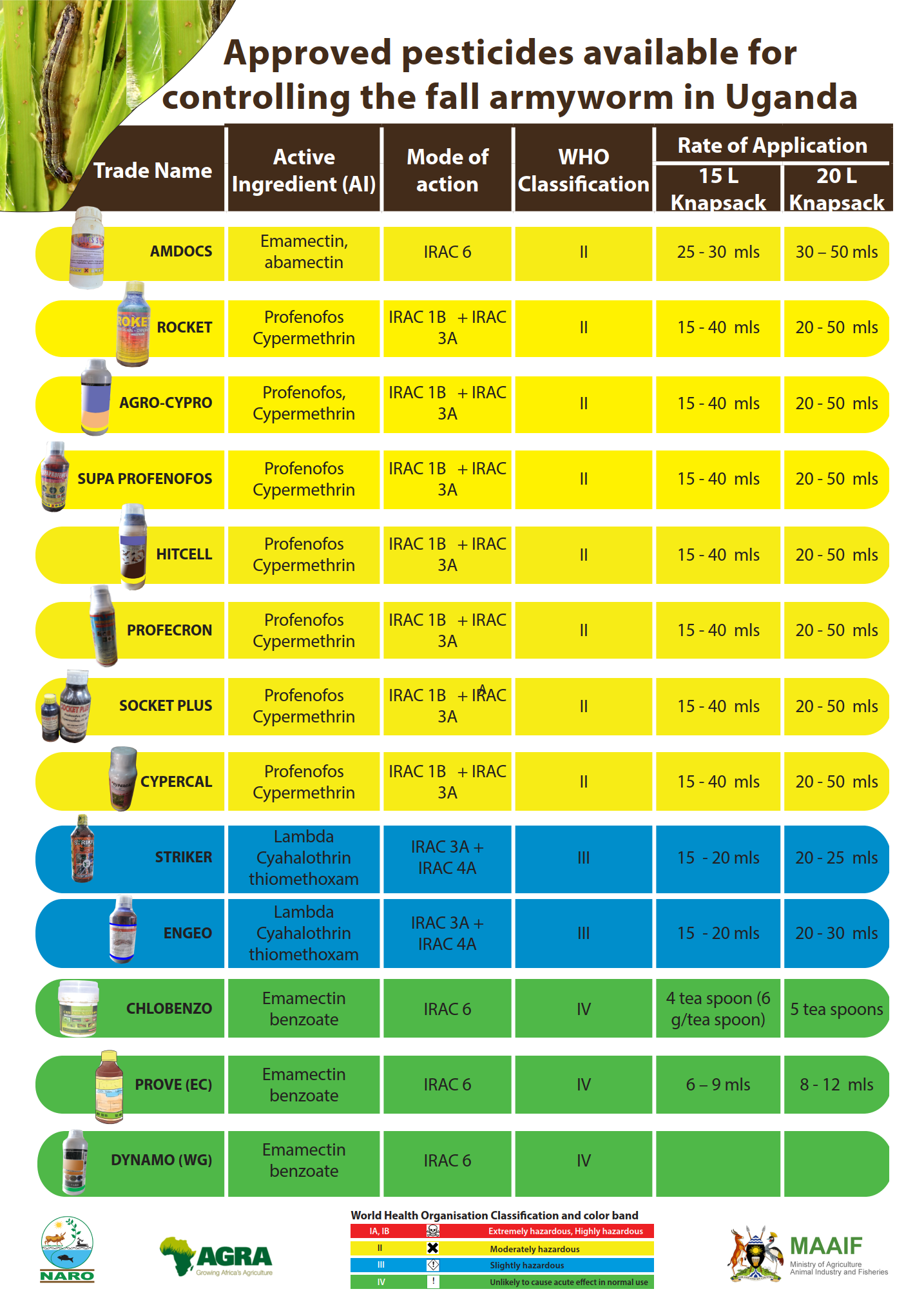


Supplementary Figure SF 3: Approved pesticides available for controlling the fall armyworm in Uganda

Supplementary Table ST 12: Suggested and purchased products during MYS

| Pesticide Brand | Suggested | | Purchased | | WHO Toxicity Class | Approved for FAW |
| --- | --- | --- | --- | --- | --- | --- |
|  | Freq | Share | Freq. | Share |  |  |
| ROCKET | 35 | 27.34% | 24 | 25.53% | II | Yes |
| STRIKER | 21 | 16.41% | 16 | 17.02% | III | Yes |
| Dudu Acelamectin | 11 | 8.59% | 8 | 8.51% | Ib | No |
| PROFECRON | 9 | 7.03% | 8 | 8.51% | II | Yes |
| DUDU-FENOS | 10 | 7.81% | 7 | 7.45% | II | No |
| Alpha Killer | 5 | 3.91% | 4 | 4.26% | II | No |
| Dudu Cyper 5% EC | 5 | 3.91% | 3 | 3.19% | II | No |
| Eminent 5 WDG | 3 | 2.34% | 3 | 3.19% | IV | No |
| DD Force | 3 | 2.34% | 2 | 2.13% | Ib | No |
| AMDOCS | 2 | 1.56% | 2 | 2.13% | II | Yes |
| Cyper Lacer | 2 | 1.56% | 2 | 2.13% | II | No |
| Cypershi 5% EC | 2 | 1.56% | 2 | 2.13% | II | No |
| Ascoris 48EC | 2 | 1.56% | 1 | 1.06% | II | No |
| Kuu Cyper | 2 | 1.56% | 1 | 1.06% | II | No |
| Lava | 2 | 1.56% | 1 | 1.06% | Ib | No |
| Ant-Killer | 1 | 0.78% | 1 | 1.06% | II | No |
| Chorpy 480 EC | 1 | 0.78% | 1 | 1.06% | II | No |
| Cyper Force | 1 | 0.78% | 1 | 1.06% | II | No |
| Lara Force | 1 | 0.78% | 1 | 1.06% | II | No |
| M-D FOS 48% EC | 1 | 0.78% | 1 | 1.06% | II | No |
| Metalamanco 72 WP | 1 | 0.78% | 1 | 1.06% | II | No |
| Supacyper | 1 | 0.78% | 1 | 1.06% | II | No |
| Tafgor 40 EC | 1 | 0.78% | 1 | 1.06% | II | No |
| TROBAN 48EC | 1 | 0.78% | 1 | 1.06% | II | No |
| Umeme | 1 | 0.78% | 1 | 1.06% | II | No |
| SOCKET PLUS | 1 | 0.78% | 0 | 0.00% | II | Yes |
| Cyclone | 1 | 0.78% | 0 | 0.00% | II | No |
| Extreme | 1 | 0.78% | 0 | 0.00% |  | No |
| SUPA PROFENOFOS | 1 | 0.78% | 0 | 0.00% | II | Yes |

FAW: Fall army worm

Supplementary Table ST 13: Original questions to Figure 5

| Column | Original Question |
| --- | --- |
| First | “We are now coming to a section where we talk about *what you say* when selling pesticides. Please answer with yes or no. Do you generally offer *any* pest and disease advice to farmers? Do you give suggestions about *which chemicals to buy* when farmers buy pesticides? Do you give any advice regarding *handling and application* of the product? Do you *explain the label* of the product? Do you mention the possibility of *health effects*? Do you give advice on *personal protective equipment*? Do you mention the possibility of *environmental effects*? Do you give advice on *storage* of the pesticide? Do you give advice on *container disposal*?” |
| Second | “We are now coming to a section where we would like to know how many of your customers ask for a *specific kind* of advice. How many of the farmers ask you for advice regarding product choice, application procedure, information on the label, health effects, PPE, environmental effects, storage of pesticide, container disposal” Answer options: None (0%), Some (25%), Half of them (50%), Most of them (75%), All of them (100%), Don't know. Displayed here: Sum of answers for 50% or more. |
| Third | “Which topics were discussed during the sales procedure *overall*?” followed by  “Who *initiated* the conversation regarding each of the following topics |
| Fourth | ”Did the agro-input dealer give you advice WITHOUT you asking?” If yes: “On what topics did you receive advice?” Probing questions: “How should I protect myself?” and “Is there any other advice you have relate to the product?” |

Supplementary Table ST 14: Label colors and areas

| Reason for coloring | % |
| --- | --- |
| Correct answer: Hazard color band | 64.2 |
| Wrong Answer: any answer not indicating hazard, risk, toxicity, etc. | 14.4 |
| Don't know | 20.9 |
| No response | 0.5 |
|  |  |
| **What color do you see?** | % |
| Red | 97.8 |
| Any other color | 0.5 |
| Don't know | 1.7 |
|  |  |
| **What is the specific meaning of this color?** | % |
| Wrong answers | 2.7 |
| General expression such as 'hazardous' or 'dangerous' | 46.0 |
| Extremely hazardous | 15.2 |
| Highly hazardous | 4.0 |
| Very Toxic | 6.2 |
| Toxic | 7.5 |
| Fatal | 1.0 |
| Don't know | 16.7 |
| No response | 0.8 |
|  |  |
| **What other colors could the label have?** | % |
| Red | 23.63 |
| Yellow | 47.76 |
| Blue | 40.3 |
| Green | 40.05 |
| Other color | 10.2 |
| Don't know | 29.35 |
| No response | 1.24 |
|  |  |
| **What do the other colors indicate?** | % |
| Wrong answers | 15.92 |
| Yellow - Moderately hazardous, harmful, toxic | 16.92 |
| Blue - Slightly hazardous, caution, (may be) harmful | 12.94 |
| Green - Unlikely to present acute hazard in normal use, not classified | 12.69 |
| Don't know | 62.44 |
| No response | 3.98 |
|  |  |
| **Please explain the difference between the two areas with similar symbols** | % |
| Wrong answers | 19.4 |
| Correct Answer: left side: 'Necessary PPE for *handling* the product',  right side 'Necessary PPE for *applying* the product' | 19.4 |
| Partially correct answer: 'Necessary PPE for the product' | 14.4 |
| Partially correct: left side: 'Necessary PPE for *handling* the product' | 4.5 |
| Partially correct: right side: 'Necessary PPE for *applying* the product' | 2.0 |
| Don't know | 38.1 |
| No response | 2.2 |

Supplementary Figure SF 4: Hazard symbol identification

Supplementary Table ST 15: Brands mentioned as best, second or third selling product.

| Brand name | n | % | Corresponding active ingredient | Group* | WHO Class |
| --- | --- | --- | --- | --- | --- |
| 2,4-D | 30 | 7.5 | 2,4- (Dimethyl) amine 720g/l | H | II |
| Ametryne | 8 | 2.0 | Ametryn 500g/l | H | II |
| Force Up | 16 | 4.0 | Glyphosate 480g/l | H | III |
| Weedmaster | 159 | 39.6 | Glyphosate 500g/l | H | III |
| Cyperlacer | 52 | 12.9 | Cypermethrin 50g/l | I | II |
| Dudu Acelamectin | 202 | 50.2 | Abamectin 1.8% + Acetamiprid 3% | I | Ib/II |
| Dudu Cyper | 74 | 18.4 | Cypermethrin 50g/l | I | II |
| Dudu Fenos | 17 | 4.2 | Profenofos 400g/l + Cypermethrin 40g/l | I | II/II |
| Lava | 61 | 15.2 | Dichlorvos 1000g/l | I | Ib |
| Profecron | 14 | 3.5 | Profenofos 400g/l + Cypermethrin 40g/l | I | II/II |
| Rocket | 187 | 46.5 | Profenofos 400g/l + Cypermethrin 40g/l | I | II/II |
| Striker | 24 | 6.0 | Lambdacyhalothrin 106g/l + thiomethoxam 141g/l | I | II/II |
| Tafgor | 56 | 13.9 | Dimethoate 400g/l | I | II |
| Dithane | 15 | 3.7 | Mancozeb 800g/kg | F | U |
| Fangocil | 13 | 3.2 | Mancozeb 640g/kg + Metalaxyl 80g/kg | F | U/II |
| Indofil | 66 | 16.4 | Mancozeb 800g/kg | F | U |
| Other | 177 | 44.0 | - | - | - |
| Don't remember | 0 | 0.0 | - | - | - |
| Don't Know | 9 | 2.2 | - | - | - |
| No response | 19 | 4.7 | - | - | - |

*Group corresponds to the chemical groups H for herbicide, I for insecticide and F for fungicide.

Supplementary Table ST 16: Corresponding active ingredients to best, second or third selling product

| Active ingredient | WHO Class | n | % |
| --- | --- | --- | --- |
| 2,4-Dichlorophenoxyacetic acid | II | 10 | 0.8 |
| Abamectin | Ib | 15 | 1.2 |
| Abamectin + Acetamiprid | Ib/II | 49 | 4.1 |
| Acetamiprid | II | 3 | 0.2 |
| Cypermethrin | II | 108 | 9.0 |
| Cypermethrin + Profenofos | II/II | 95 | 7.9 |
| Dichlorvos | Ib | 37 | 3.1 |
| Dimethoate | II | 46 | 3.8 |
| Glyphosate | III | 121 | 10.0 |
| Lambda cyhalothrin | II | 5 | 0.4 |
| Lambda cyhalothrin + Thiamethoxam | II/II | 5 | 0.4 |
| Mancozeb | U | 47 | 3.9 |
| Profenofos | II | 20 | 1.7 |
| Thiamethoxam | II | 1 | 0.1 |
| Other | - | 24 | 2.0 |
| Don't remember | - | 38 | 3.2 |
| Don't Know | - | 555 | 46.1 |
| No response | - | 26 | 2.2 |
| Total |  | 1205 | 100 |

Supplementary Figure SF 5: Information sources of farmers according to agro-input dealers; best* indicating: "the best way to inform farmers about safe pesticide use". All options were read out.

Supplementary Table ST 17: Agro-input dealers’ attitudes regarding license, counterfeits and management of pest resistance

| Do you consider the license relevant? (%Yes) | 88.1 |
| --- | --- |
| Why? | % |
| Enables business according to regulation | 50.8 |
| Enables tax payment | 20.7 |
| Quality assurance to the customer | 19.9 |
| Enables occupational safety | 19.9 |
| Enables Business Promotion | 5.0 |
| Other | 1.7 |
| Don't Know | 5.7 |
| No response | 4.2 |
|  |  |
| 119) What are the biggest problems with counterfeits? | % |
| They are less or not effective | 73.9% |
| They negatively impact the farmer's business | 55.5% |
| They negatively impact the agro-dealer's business | 45.3% |
| They negatively impact human health | 14.4% |
| They negatively impact on the environment | 10.4% |
| Other | 1.6% |
| Don't know | 0.5% |
| No response | 0.3% |
|  |  |
| 121) What do you do in your business to prevent and manage pest resistance? | % |
| Better advising the farmer | 33.8% |
| Recommending stronger pesticides to the farmers | 23.6% |
| Better consulting with the supplier | 19.9% |
| Buying more specific (targeted) pesticides from suppliers | 14.9% |
| Buying different pesticides from the suppliers (pesticide rotation) | 13.4% |
| Recommending different pesticides to the farmers (pesticide rotation) | 11.9% |
| Recommending more specific (targeted) pesticides to the farmers | 11.4% |
| Buying stronger pesticides from the suppliers | 9.0% |
| Other | 1.7% |
| Don't know | 0.7% |

Supplementary Table ST 18: Alternatives to synthetic pesticides and their limitations

|  | % |
| --- | --- |
| **Agro-input dealers aware of alternatives to synthethic pesticide pest management** | **78.4** |
|  |  |
| **Alternative options** | **%** |
| Cultural/ ecological (sanitation, tillage, crop spacing, crop rotation, push-pull) | 58.9 |
| Chemical (biopesticides / natural pesticides / organic pesticides) | 36.3 |
| Biological (release/promotion of natural enemies) | 27.1 |
| Mechanical (hand picking of insects or weeds, protective covers like insect nets) | 25.2 |
| Host plant resistance (crop variety less vulnerable to pest attack) | 6.7 |
| Behavioral (pheromone/hormone traps) | 5.7 |
| Other | 0.6 |
|  |  |
| **Limitations to alternative options** | **%** |
| Less effective against pests | 53.8 |
| Time consuming / Labour intensive | 47.8 |
| More expensive | 14.3 |
| Knowledge and skill demanding | 12.1 |
| Materials not readily available | 11.1 |
| Difficult to mix | 6.4 |
| Can't be easily used on large scale | 5.7 |
| Smell from materials | 1.9 |
| Mainly preventative than curative | 1.3 |
| Some irritate eyes and skin | 1.3 |
| Other | 4.5 |
| Don't know | 4.5 |
| No response | 1.6 |

Supplementary Figure SF 6: Comparison of synthetic pesticides with alternatives to them

Supplementary Table ST 19: Recommendations and corresponding reasons

|  | n | Yes (%) | No (%) |
| --- | --- | --- | --- |
| Recommending pesticide use over alternative strategies | 402 | 68.7 | 31.3 |
| Reasons for recommendation |  |  |  |
| Synthetic pesticides are more effective and work faster | 200 | 90.5 | 9.5 |
| For economic reasons (time, money) | 112 | 92.9 | 7.1 |
| To protect the human health | 100 | 20.0 | 80.0 |
| To protect the environment (e.g., sustainability) | 73 | 21.9 | 78.1 |
| Because it is more practical and easy | 51 | 96.1 | 3.9 |
| Source of income | 10 | 100.0 | 0.0 |
| Alternatives not known/available | 9 | 88.9 | 11.1 |
| Higher Yield | 7 | 100.0 | 0.0 |
| For cultural or traditional reasons | 4 | 50.0 | 50.0 |
| Other | 4 | 75.0 | 25.0 |
| Don't know | 2 | 50.0 | 50.0 |

Supplementary Table ST 20: Symptoms of pesticide poisoning recalled (known) or experienced.

|  | Experienced (%) | Known (%) | Ratio |
| --- | --- | --- | --- |
| Skin irritation | 22.4 | 57.2 | 0.39 |
| Headache | 29.1 | 44.0 | 0.66 |
| Itchy eyes | 11.4 | 37.3 | 0.31 |
| Vomiting | 5.7 | 33.3 | 0.17 |
| Respiratory difficulties | 23.6 | 29.1 | 0.81 |
| Abdominal pain | 7.5 | 25.1 | 0.30 |
| Dizziness | 11.2 | 19.9 | 0.56 |
| Nausea | 11.7 | 19.2 | 0.61 |
| Other | 4.7 | 17.2 | 0.28 |
| Muscular weakness | 6.0 | 9.2 | 0.65 |
| Chest pain | 5.0 | 7.5 | 0.67 |
| Extreme tiredness | 5.5 | 6.7 | 0.81 |
| Blurred vision | 2.2 | 4.2 | 0.53 |
| Dry mouth | 2.0 | 3.0 | 0.67 |
| Back pain | 2.0 | 3.0 | 0.67 |
| Salivation | 0.7 | 2.7 | 0.27 |
| Loss of appetite | 2.2 | 2.7 | 0.82 |
| Excessive sweating | 2.0 | 2.5 | 0.80 |
| Trembling hands | 1.2 | 1.7 | 0.71 |
| Lack of coordination | 0.7 | 1.2 | 0.60 |
| Speech difficulty | 0.2 | 1.0 | 0.25 |

Supplementary Table ST 21: Through which body parts do you think pesticides can enter us?

| body party entry site | % |
| --- | --- |
| Nose (inhalation) | 92.5 |
| Skin (dermal) | 88.3 |
| Mouth (ingestion) | 78.4 |
| Eyes (mucous membranes) | 60.4 |
| Ears | 28.9 |
| Other | 1.5 |
| Don't know | 0.5 |
| None | 0.2 |

Supplementary Table ST 22: Pesticide trends over the past and future five years within community

|  | **Increasing** | **Constant** | **Decreasing** | **Don't know** |
| --- | --- | --- | --- | --- |
| past | 91.0 | 2.5 | 3.0 | 3.5 |
| future | 86.8 | 1.5 | 5.2 | 6.5 |

Supplementary Table ST 23:Reasons for pesticide trends

| Can you give a reason for this trend? |  |
| --- | --- |
| Number of farmers increased/decreased | 31.1 |
| Pesticides are required to obtain good/any harvest at all | 22.1 |
| Abundance of pest organisms increased/decreased | 14.9 |
| Pesticides reduce labour | 8.0 |
| Pesticides are effective | 3.7 |
| Other | 3.2 |
| Organic farming increases/decreases | 2.5 |
| Pesticides are advertised/farmers are a | 2.0 |
| Pesticides increase yield | 1.7 |
| Agriculture modernizes | 1.7 |
| Farmers are sensitized about negative e | 1.7 |
| Don't Know | 1.7 |
| Pesticides are cheaper | 1.5 |
| Weather / Climate Change | 1.2 |
| soils aren't fertile | 1.0 |
| Farms are bigger | 0.8 |
| Counterfeits increase | 0.5 |
| No response | 0.5 |


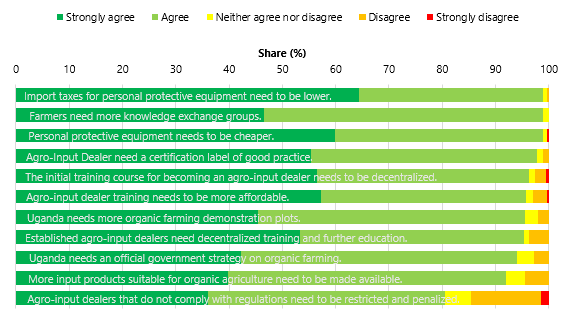


Supplementary Figure SF 7: Attitudes regarding future possible change in the pesticide sector in Uganda.

Supplementary Table ST 24: What companies are you subscribed to receive regular messages with business-related information on your mobile phone?

| Bukoola Chemicals Industries Ltd | 35.8% |
| --- | --- |
| Wefarm | 22.8% |
| various verified Agrodealers | 21.6% |
| East African Seed (U) Ltd | 14.2% |
| Daps Distribution Co.Ltd | 11.1% |
| various unverified Agrodealers | 11.1% |
| Jubilee Insurance Company of Uganda Ltd | 9.9% |
| NGOs and Government | 6.8% |
| No response / Don't remember / Don't know / Unrelated answers | 7.4% |

Supplementary Table ST 25: Detailed safety equipment layout

| Is there any safety equipment available for staff? | % |
| --- | --- |
| nothing available (not visible) | 61.2 |
| hat | 3.1 |
| goggles or face shields for eye and face protection | 4.6 |
| specific or all-purpose gas masks | 9.4 |
| respirators | 9.4 |
| long-sleeved, buttoned coat or suit completely covering the worker | 11.2 |
| gloves (water-proof and impervious) | 18.1 |
| boots | 15.6 |
|  |  |
| **Which of the following facilities are available in the shop to wash eyes or remove toxic materials from the skin?** | **%** |
| nothing available (not visible) | 41.1 |
| facilities for washing eyes such as fixed or portable eye-wash fountains. | 0.5 |
| adequate emergency water supply for washing off corrosive or toxic materials getting on the skin | 0.5 |
| Water Bucket | 42.6 |
| Soap / detergent | 29.9 |
| Tap Water outside shop | 15.6 |
| Tap Water inside shop | 8.7 |
|  |  |
| **Which of the following materials are available to cleanup and decontaminate spills?** | **%** |
| nothing available (not visible) | 41.8 |
| broom | 56.6 |
| inert absorbent material such as sand, soil or sawdust | 1.3 |
| disposable container | 2.3 |
| hydrated lime or soda ash | 0.3 |
| clay or similar material for absorbing scrubbing liquid | 1.8 |
